# Distal tip cell migration mutants of *Caenorhabditis elegans* are rescued by bioequivalent outputs from chondroitin and N-glycosylation pathways

**DOI:** 10.1016/j.jbc.2025.110895

**Published:** 2025-11-04

**Authors:** James W. Dennis, Robert J. Dunn, Naomi Levy-Strumpf, Maria Brooun, Nadeem Moghal, Joseph G. Culotti

**Affiliations:** 1 Lunenfeld-Tanenbaum Research Institute of Mt. Sinai Hospital, Toronto, Canada; 2 Department of Molecular Genetics, University of Toronto, Toronto, Canada; 3 Cell and Systems Biology, University of Toronto, Toronto Canada; 4 Princess Margaret Cancer Centre/University Health Network, Department of Medical Biophysics, University of Toronto, Toronto, Ontario, Canada

**Keywords:** cell migration, *C. elegans*, chondroitin, N-glycans, glycosyltransferases compensation, β-galactoside

## Abstract

In *Caenorhabditis elegans,* gonadal morphogenesis begins with the post-embryonic birth of two distal tip cells (DTCs) that migrate in phase 1 along the ventral body-wall in opposite directions. Phase 2 comprises a 90^°^ turn followed by migration in a dorsal direction. Here, we identify a genetic dependency between two glycosylation pathways that modify proteins at different sites and display bioequivalence that ensures phase 2 navigation at elevated temperatures. Loss-of-function (*lf*) mutations in one putative and one proven β4N-acetylgalactosaminyltransferase:- *ngat-1(null)* predicted to affect the formation of LacdiNAc on complex-type N-glycans and *mig-22(lf)* affecting chondroitin synthesis, each disrupted phase 2 with incomplete penetrance, whereas *ngat-1;mig-22* double mutant was disrupted with high penetrance (>90%). Single mutants were rescued by a 1- to 2-day starvation-induced larval diapause (protective developmental arrest) followed by refeeding (S/R), but *ngat-1;mig-22* was not rescued, suggesting the two gene products function redundantly. GlcNAc supplementation to the hexosamine biosynthesis pathway (HBP), or gain-of-function *mig-22(k185gf)* rescued *ngat-1(null),* consistent with a compensating redundancy driven by enhanced UDP-HexNAc flux. Our results suggest that β-galactosides, structural features common to chondroitin and glycoproteins, contribute robustness that ensures DTC phase 2 fidelity under stressful thermal and nutritional conditions.

Directed migration of cells and pioneer axons are conserved processes widely important for animal development, as well as cancer invasion and metastasis. *Caenorhabditis elegans* is an ideal genetic model to visualize defects and study the genes required for migration of individual cell types at different stages of development (1). Mutations in genes encoding basement membrane (BM) glycoproteins and proteoglycans have been shown to disrupt *C. elegans* DTC migration, often with partial penetrance (2, 3, 4, 5, 6, 7). In the first larval stage (L1), two gonadal DTCs are born and migrate longitudinally along the ventral body-wall muscles (BWMs) in opposite directions away from mid-body, with an attached tubular gonad arm forming behind. In phase 2, each DTC turns 90° to migrate across the lateral epidermal BM in a ventral to dorsal direction. Phase 3 comprises another 90° turn and migration along the dorsal BWMs, returning to the midbody. The shape of each gonad arm represents the path taken by DTC migration. BM glycoproteins display overlapping structural features, which may have evolved to ensure high fidelity during morphogenesis in diverse environmental conditions. Here, we identify genetic redundancies that extend tolerance to thermal and nutritional variation for accurate DTC navigation.

Classic studies have shown that the BM glycoprotein UNC-6/Netrin is required for DTC phase 2 migration by interacting with UNC-5 and UNC-40/DCC receptors on DTCs (1). UNC-6, and its receptors are also required for axon guidance (6, 8). The phase 2 defects in *unc-5*, *unc-6* and *unc-40* single mutants frequently fail to initiate the first turn, but if phase 2 is initiated, DTC migration follows a normal ventral to dorsal trajectory before returning to the mid-body on the dorsal side. Thus UNC-6 acting redundantly with Wnt and its receptors (9), are required to sense and initiate the turn (1, 6), while additional cues in the BM are necessary to guide and complete phase 2 migration. These include additional glycoproteins; the ADAM metalloproteases, GON-1 and MIG-17 (10, 11), integrins INA-1 and PAT-3 (12, 13) as well as proteoglycans *mig-6*/CGP-17/papilin (4), *unc-52*/perlecan (14) and *sdn-1*/syndecan (15) plus genes encoding support for N-glycosylation and chondroitin biosynthesis including; *mig-23*/nucleotide diphosphatase required for Golgi import of sugar-nucleotides (11), *cogc-1* and *cogc-3* required for glycoprotein trafficking through the Golgi (16), *gale-1/*UDP-galactose-4-epimerase which maintains equilibrium between UDP-Glc(NAc) and UDP-Gal(NAc) (17) and two chondroitin synthetases *sqv-5* (18, 19) and *mig-22* (20). SQV-5 and MIG-22 both have dual activities that transfer from UDP-GalNAc and UDP-GlcA to generate the repeating GalNAcβ1-4GlcAβ1-3 units of the linear chondroitin polymer (21) (Fig. 1, *A* and *B*).

**Figure 1:**
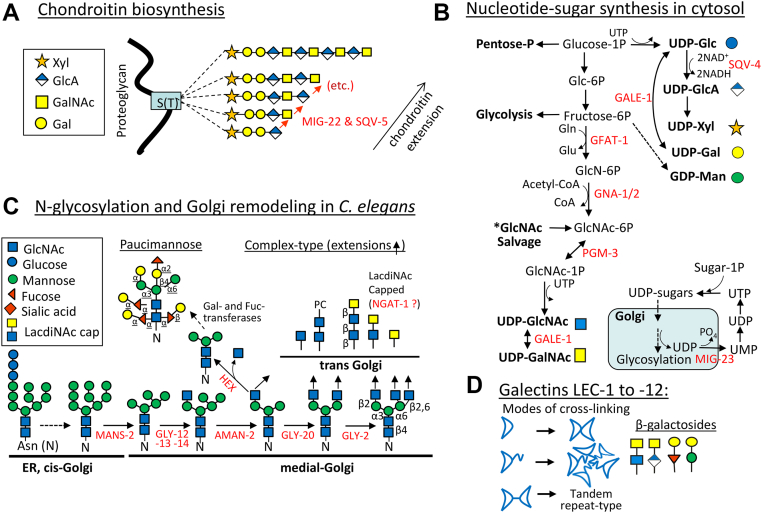
**Outline of pathways and glycan structures in this report.***A*, Chondroitin biosynthesis is initiated in the ER and chains are extended in the Golgi. *B*, the hexosamine biosynthesis pathway (HBP) supplies the rate-limiting substrate, UDP-GlcNAc, to the MGAT enzymes of the N-glycan branching pathway. GALE-1 epimerase converts UDP-GlcNAc to UDP-GalNAc as required for biosynthesis of chondroitin, glycolipid, and glycoproteins. *C*, co-translational N-glycosylation of proteins in the secretory pathway of vertebrates and *C. elegans* is conserved, but remodeling in cis/medial Golgi differs after the action of MGAT-1 (encoded by *gly-12*, *gly13* and *gly-14)*. A portion becomes paucimannose by the action of HEX β-hexosaminidase and α-mannosidases (*solid arrow*), while the rest become GlcNAc-branched complex-type glycans, either unsubstituted, capped by a β4-linked GalNAc or phosphorylcholine (PC). In the MGAT-1 triple mutant, complex-type N-glycans are absent, and the MGAT-1 precursor is redirected to a subset of paucimannose structures (27). *D*, The GalNAcβ1-4GlcA repeats in chondroitin, and the GalNAcβ1-4GlcNAc (LacdiNAc) product of NGAT-1 in complex-type N-glycans are potential ligands for galectins. LEC −1, −2, −3, −4, −5 are tandem repeat-type galectins with two carbohydrate recognition domains (CRD). LEC −6, −7, −8, −9, −10, −11 are prototype galectins with one CRD, and some with intrinsically disordered regions (IDR) as in mammalian galectin-3.

We reported previously that *ngat-1* deletion and truncation mutants *(ev821* and *ev823)* display temperature-sensitive posterior DTC phase 2 migration defects and very few anterior DTC defects (22). *ngat-1* (W02B12.11) encodes a homolog of *bre-4* in the GT7 family of β1,4 Gal- and GalNAc- transferases, and both protein sequences have catalytic sites characteristic of β1,4 GalNAc-transferase activity (23). Unlike *ngat-1* mutations, *bre-4(ok3167)* loss-of-function does not affect DTC migration (22). Whereas BRE-4 has proven β1,4 GalNAc-transferase enzyme activity (24), the above-noted homology suggests that NGAT-1 has similar activity involved in Golgi N-glycan modifications. Genetic evidence also suggests that NGAT-1 is a β1,4 GalNAc-transferase involved in Golgi N-glycan modifications (22), following the action of N-acetylglucosaminyltransferase I (MGAT1). In *C*. *elegans,* MGAT-1 activity is encoded by three paralogues, *gly-14, gly-12* and *gly-13* (25), which catalyze the addition of GlcNAc, producing GlcNAcβ1-2Man_5_GlcNAc_2_-Asn (Fig. 1*C*). Trimming by α-mannosidase II yields GlcNAcβ1-2Man_3_GlcNAc_2_-Asn, allowing further GlcNAc-branching by MGAT-2 (*gly-20*) and MGAT-5 (*gly-2*). These branched N-glycans can be modified by β1,4-linked GalNAc to form LacdiNAc motifs. The *gly-14;gly-12;gly-13* triple KO MGAT-1 mutant, and the *ngat-1* mutants displayed a similar temperature sensitivity and penetrance for posterior DTC phase 2 defects, as well as low penetrance in the anterior (Table S1). Importantly, defects were not enhanced in a *ngat-1*;*gly-14 gly-12;gly-13* quadruple mutant, consistent with NGAT-1 and MGAT-1 functioning in the same pathway (22). We propose that NGAT-1 is an MGAT-1-dependent enzyme forming LacdiNAc (GalNAcβ1,4GlcNAc) previously described on a subset of *C. elegans* complex-type N-glycans (26).

Golgi β-hexosaminidases (HEX) remove GlcNAc from a subset of the GlcNAcβ1-2Man_3_GlcNAc_2_-Asn intermediate, creating paucimannose (*i.e.* Man_3_GlcNAc_2_-Asn), which undergoes further substitutions of fucose and galactose, producing N-glycan structures not found in vertebrates (26, 27). Galα/β1-4Fuc- and Galα/β1-4Man-sequences are found on paucimannose N-glycans (28, 29), while GalNAcβ1-4GlcNAc- (LacdiNAc) is only on complex type N-glycans (Fig. 1*C*). These are β-galactosides, a subset of disaccharides with either Gal or GalNAc in a β1 linkage to another monosaccharide (30). β-galactosides are ligands for galectins*,* a conserved family of glycan-binding proteins (31) involved in many physiological functions such as inflammation, cell migration, endocytosis and cytokine signaling (32, 33) (Fig. 1*D*).

Chondroitin is a repeating polymer of β-galactoside epitopes (GalNAcβ1-4GlcAβ1-3) and also a ligand for *C. elegans* and mammalian galectins (34, 35). Chondroitin synthesis and N-glycan remodeling are independent pathways producing glycans of varied sizes, branching and positions on proteins. Glycoproteins represent ∼25% of the proteome, and include transmembrane receptors, solute transporters and secreted glycoprotein, while chondroitin proteoglycans are limited to ∼40 to 60 members that mediate biophysical interactions in tissues and regulate growth factor availability in BMs (36). Genetic evidence suggests that NGAT-1 modification of complex-type N-glycans with LacdiNAc is required for DTC phase 2 navigation at high growth temperatures (22). Here, we report that the *ngat-1* phase 2 defects at high growth temperatures are rescued by GlcNAc supplementation, the gain-of-function mutation *mig-22(k185gf)* (37)*,* or starvation-induced L1 diapause (∼24–48h) followed by refeeding (S/R). Synergistic enhancement in the *ngat-1;mig-22* double mutant revealed a genetic redundancy that was not rescued by S/R or GlcNAc supplementation, indicating reciprocal compensation for each single mutant defect by the non-mutant gene. Additional data using the gain-of-function *gfat-1(dh468gf)* mutant (38) supports a model by which increased UDP-GlcNAc flux through the Golgi N-glycan remodeling and chondroitin biosynthesis pathways ensures DTC phase 2 fidelity under temperature and nutrient stresses. Based on these findings, we propose a model, where the bioequivalence of β-galactosides on chondroitin proteoglycans and the N-glycans on glycoproteins allows galectin-mediated clustering of cues and receptors at the cell surface required to drive phase 2 migration.

## Results

### Phenotyping of *ngat-1(ev840*) null and other DTC phase 2 mutants

**Figure 2:**
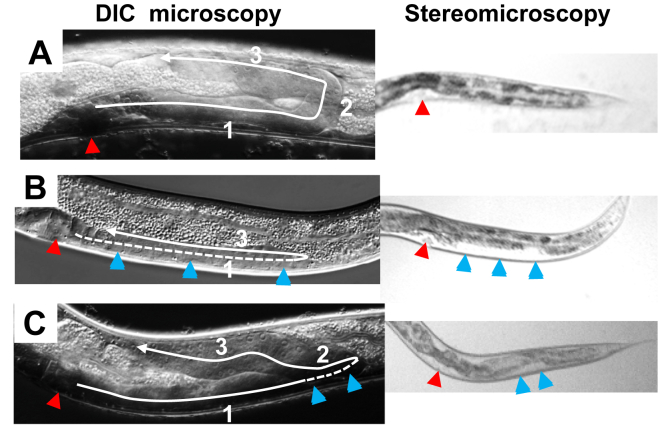
**Posterior Distal Tip Cell (DTC) trajectories.***A*, WT (*top left*) and *(B, C)* examples of *ngat-1(840)* mutant DTC phenotypes in hermaphrodite L4 animals grown at 25 °C. Lateral view of the posterior gonad arm in L4 stage hermaphrodites by differential interference contrast microscopy (DIC, *left* panels) and stereomicroscopy (*right* panels). Dorsal is up and anterior is left in all panels. The shape of each gonad arm reflects the trajectory of each migrating DTC (*white line*), beginning at the vulva (*red arrowhead*). *A*, A gonad arm displaying the 3-phase wild-type trajectory observed in some *ngat-1* mutant animals. Note the ventral to dorsal migration of phase 2. *B*, *ngat-1* mutant animal in which phase 2 fails, and the DTC returns to mid-body along approximately the same path (*solid line*) it took in phase 1 (*dashed line*). The overlap between the two portions of the trajectory pushes the opaque intestine dorsally and forms a ventral clear patch of side-by-side translucent gonad arms observable by stereomicroscopy (*blue arrowheads*). *C*, phase 1 (*solid then dashed line*) is normal, but in phase 2 the DTC has begun to return to mid-body on the ventral side before turning dorsally. The dashed line portion of phase 1 represents the overlap with phase 2 migration and accounts for the small ventral clear patch in the *right* panel.

**Figure 3:**
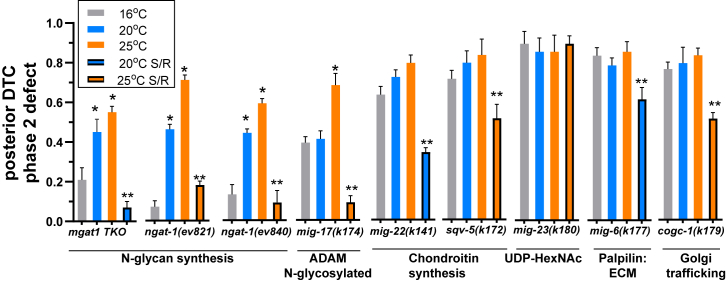
**Posterior DTC phase 2 defects: penetrance, temperature sensitivity, and rescue by L1-starvation/refeeding (S/R).** Single mutants and the *mgat-1* triple null mutant which removes three paralogues *(gly-14;gly-13;gly-12)* were fed *ad libitum (AL)* at 16 °C, 20 °C and 25 °C and scored for phase 2 DTC migration defects. The fourth bar represents defects after early L1 larval starvation for a minimum of 24h followed by refeeding (S/R) and data being collected one or 2 days later in L4 and young adults as noted in Figure 2 (Table S1). Statistical comparisons between mutants at 16 °C and elevated temperatures (∗*p* < 0.05) and rescue by S/R at elevated temperatures (∗∗*p* < 0.05). In *N2* animals, posterior phase 2 defects were not observed in *AL* fed at 16 °C; whereas 6/326 (2%) scored at 25 °C, and no defects (0/85) in S/R conditions at 25 °C (Table S1*B*).

DTC phase 2 migration defects cause a clear patch on the ventral side of the worm, detected in L4 and young adults by low magnification stereomicroscopy and confirmed by differential interference contrast microscopy (Fig. 2). The alleles *ngat-1(ev821)* and *ngat-1(ev823)* are partial deletions of the gene (22). *ngat-1(ev821)* displayed more penetrant posterior DTC phase 2 migration defects than the *ngat-1(ev823)* allele (22). *ngat-1(ev821)* is a premature stop codon predicted to terminate translation near the active center of the encoded NGAT-1 enzyme. This should eliminate enzymatic activity, but the predicted residual peptide may have antagonistic effects on DTC phase 2 migration. To better quantify the null phenotype, we used CRISPR CAS-9 mutagenesis and isolated the *ngat-1(ev840)* allele with a deletion of the protein-coding DNA except for 13 codons at the N-terminus. Penetrance of the posterior DTC defects in *ngat-1(ev840)* fed *ad libitum* (*AL)* at 25 °C was 59% (296/501) (Fig. 3, Table S1), significantly lower than *ngat-1(ev821)* at 71% (310/439) (*p* = 0.0002). The results are consistent with *ev821* having an additional antagonistic effect on DTC phase 2 migration at 25 °C, perhaps due to the truncated mutant NGAT-1 protein. All three alleles are recessive, maternally rescued (not shown), and exhibit temperature-sensitive defects in posterior DTC phase 2 migration, with little effect on anterior migration. *ngat-1(ev823)* and *ngat-1(ev840)* display very similar phenotypes and were used in subsequent experiments.

DTC phase 2 migration mutants of interest were compared for temperature sensitivity. These include *mig-17(k174)*, *cogc-1(k179)*, *mig-23(k180)* and the *mgat-1* triple – all believed to be null alleles, and the hypomorphic *mig-22(k141)*, *sqv-5(k172)*, *mig-6(k177)* alleles for which null alleles are lethal. Phase 2 DTC defects (posterior, unless noted otherwise) increased significantly in the *mgat-1* triple, *ngat-1* and *mig-17* mutants when grown at increasing temperatures from 16 °C to 25 °C (Fig. 3, Table S1). High penetrance defects in *cogc-1*, *mig-6*, *mig-23 mig-22* and *sqv-5* mutants at 16 °C did not change significantly with increased growth temperature, although *mig-22* and *sqv-5* showed a small increase (Fig. 3). We also noted that anterior DTC phase 2 migration defects in the *cogc-1* mutant were increased at low temperature (Table S1).

### Rescue of DTC phase 2 defects by starvation-induced L1 diapause and re-feeding (S/R)

After thawing a frozen stock of *ngat-1(ev821)*, we noted by stereomicroscopy that very few animals grown from frozen stocks had a ventral clear patch (VCP), an indicator of the phase 2 DTC migration defect (Fig. 2) (22). Since frozen stocks are made from starved animals, this observation suggested that 1- to 2-day starvation followed by refeeding may stimulate parallel pathways supplying glycoconjugates that compensate for ones missing in the *ngat-1* mutant. In preliminary experiments with *ngat-1(ev821)* and *mig-22(k141)* mutants, animals were grown until *E. coli* food source disappeared, causing animals to form Dauer larvae before being refed. The penetrance of DTC phase 2 defects was 2% (2/84) for refed *mig-22(k141)* mutant dauers compared to 72% (219/304) when fed *AL,* and defects of 13% (15/118) for refed *ngat-1(ev821)* mutant dauers compared to 71% (310/439) when fed *AL* (*p* < 0.0001) (Table S1). Experiments with dauers were not pursued; instead, hatchlings on nematode growth media plates lacking *E. coli* as food were incubated for a minimum of 24 h before re-feeding (S/R), then L4 and young adults were scored for DTC phase 2 defects.

*ngat-1(ev821)* worms fed *AL* at 20°C displayed 48% DTC phase 2 defects and only 2% following S/R. At 25 °C, penetrance was 71% when fed *AL* and 18% following S/R (Fig. 3, Table S1). Similarly, the *ngat-1(ev840)* phase 2 defects fed *AL* at 25 °C were 59% and reduced to 6% by S/R. The *mgat-1* triple mutant fed *AL* at 25 °C was rescued by S/R from 55% to 7% defects. Defects in *ngat-1(ev840)* and *mgat-1* triple mutants displayed a high temperature sensitivity which was largely rescued by S/R, whereas the *mig-17(k174)* mutant was more penetrant at 16^o^C and less temperature sensitive. Phase 2 defects in *mig-22*, *sqv-5, mig-23, mig-6* and *cogc-1* mutants displayed a higher penetrance at 16 °C and did not change significantly with increased growth temperature. Only *mig-23* was completely resistant to rescue by S/R. *mig-23* encodes a nucleoside-diphosphatase that converts UDP to UMP for export from the Golgi in exchange for UDP-sugar import, where chondroitin synthesis and N-glycan remodeling occur (39). The *mig-23(k180)* mutation inhibits both pathways required for the fidelity of DTC phase 2 migration, as confirmed by experiments below (Fig. 3, Table S1).

### Compensating redundancies by NGAT-1 and MIG-22 ensures high fidelity DTC phase 2 migration

**Figure 4:**
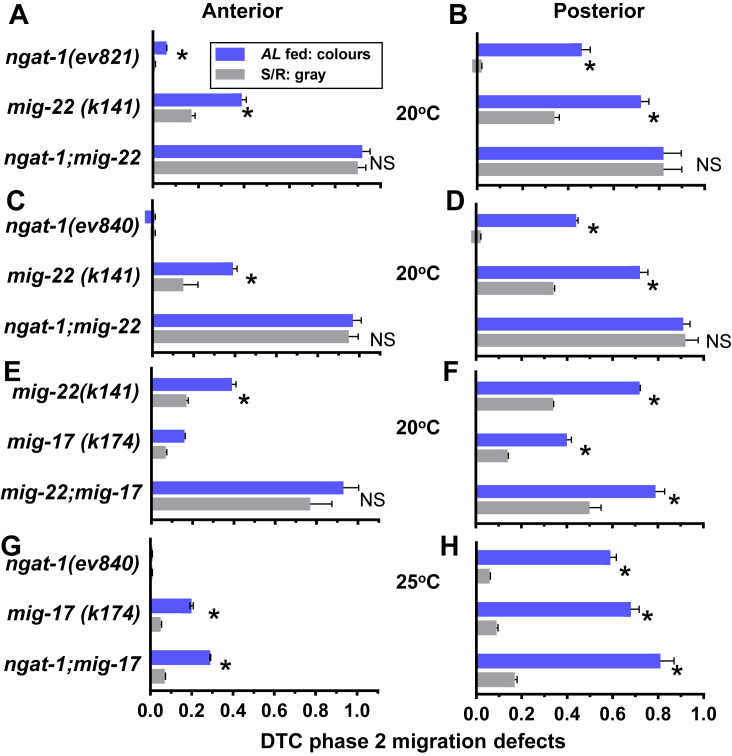
**DTC phase 2 defects in single and double mutants fed *AL* or following S/R.** Single and double mutants *(A, B) ngat-1(ev821), mig-22(k141)*, *(C, D) ngat-1(ev840), mig-22(k141)*, *(E, F) mig-22(k141), mig-17(k174)* and *(G, H) ngat-1(ev840), mig-17(k174).* The mutants were fed *AL* for greater than one generation at the indicated temperature, or starvation as L1 larvae for a minimum of 24 h followed by refeeding (*gray bars*). Anterior (*left*) and posterior (*right*) phase 2 defects were scored in L4 larvae and young adults as noted in Figure 2 (Table S2). Temperature sensitivity differed for the single mutants (see Fig. 3). *ngat-1* at 20 °C was sufficient to induce DTC phase 2 defects, and necessary for double mutant experiments with *mig-22* (Fig. 1, *A*–*D*), as temperatures above 20 °C reduced viability of *mig-22*. Conversely, heat sensitivity of *mig-17* is observed only above 20 °C, and therefore, the *ngat-1;mig-17* double was done at 25 °C (Fig. 1, *G* and *H*), where both mutations are viable and DTC defects are readily displayed. Statistical comparisons between *AL* and S/R ∗ *p* < 0.05.

Single mutants had fewer defects in the anterior relative to posterior, suggesting residual enzyme activities were normally higher in the anterior. Double mutants of *ngat-1, mig-22* and *mig-17* were made in all combinations and phase 2 defects quantified in *AL* fed and S/R conditions at elevated temperature. The incidence of anterior phase 2 defects increased synergistically in *ngat-1;mig-22* and *mig-22;mig-17* double mutants relative to the single mutants (Fig. 4, *A* and *C*, Table S2), suggesting that genes pairs function redundantly in the anterior. Phase 2 defects in both the anterior and posterior of *ngat-1;mig-22* reached an upper limit of ∼90%, suggesting the N-glycan and chondroitin products of NGAT-1 and MIG-22 are necessary in aggregate for phase 2 navigation by both DTCs (Fig. 4, *A*–*D*). In *mig-22;mig-17*, phase two defects also displayed additivity and high penetrance in the anterior and posterior (Fig. 4, *E* and *F*). Posterior defects in *ngat-1;mig-17* were enhanced relative to the single mutants; however, anterior defects in the double mutant remained low (29%), suggesting a greater requirement for NGAT-1 in the posterior to modify glycoproteins other than MIG-17 (Fig. 4, *G* and *H*).

In single mutants, rescue of posterior defects by S/R was 93% for *ngat-1(k840)* (from 44% to 3%)*,* 53% for *mig-22* (72% to 34%) and 66% for *mig-17* (41% to 14%) (Fig. 4, *B* and *D*). S/R rescued posterior defects in *ngat-1;mig-17* (81% to 17%) and partially rescued *mig-22;mig-17* (79% to 50%) (Fig. 4, *H* and *F*), but failed to rescue *ngat-1;mig-22* (91% to 92%) (Fig. 4, *A* and *B*). The synergistically high penetrance observed for the *ngat-1;mig-22* and the complete failure to rescue by S/R suggest that NGAT-1 and MIG-22 enzyme products function redundantly in a mutually compensating manner for S/R rescue **(**gray bars in Fig. 4, *A*–*D*). β-galactosides are the only apparent structural similarity common to glycoproteins and chondroitin proteoglycans that might account for this functional redundancy (34, 35). We propose that β-galactosides contribute to the bioequivalence previously reported within the complex-type N-glycans in mammals (40, 41), extended herein to chondroitin proteoglycans.

### Rescues by GlcNAc supplementation and gain-of-function mutations

**Figure 5:**
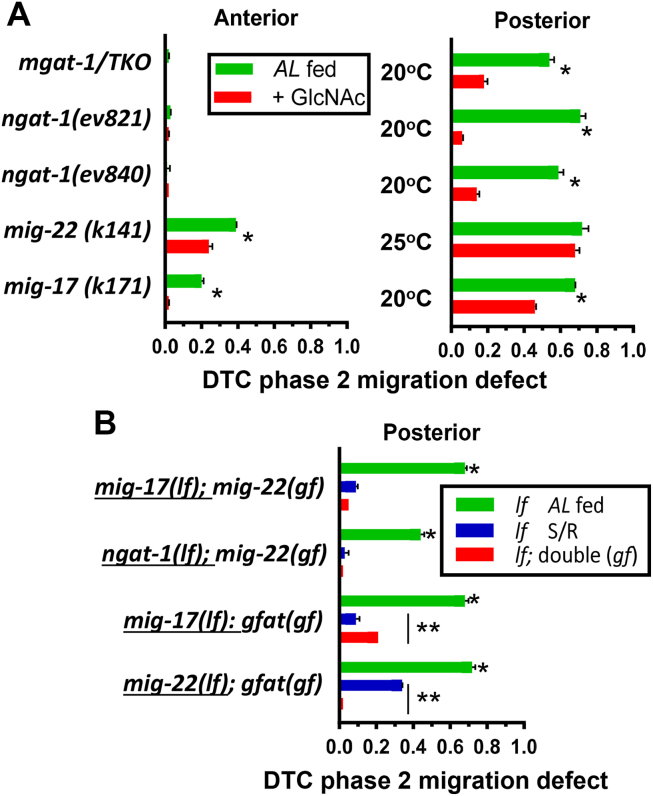
**GlcNAc supplementation and doubles with gain-of-function mutants.***A*, penetrance of posterior DTC phase 2 defects in fed *AL* at 20 °C with and without 7 to 10 mM GlcNAc supplement (Table S3). *B*, loss of function mutants in *AL* fed and S/R conditions and as double mutants with *mig-22(gf)* or *gfat(dh468gf)* scored for defects as noted in Figure 2 (Table S4). Statistical comparisons with *AL* fed ∗*p* < 0.05 and pairs as indicated ∗∗*p* < 0.05.

Denzel *et al.* (38). screened for resistance to ER stress (tunicamycin treatment) in *C. elegans* and isolated three gain-of-function mutants in *gfat-1,* encoding glutamine fructose-6-phosphate aminotransferase, the first committed step in HBP (Fig. 1*B*). The *gfat-1(gf)* mutants extend lifespan in a manner that requires constitutive levels of *ire-1* and *xbp-1*, increased ER-associated protein degradation (ERAD) and autophagy. Supplementing the diet with GlcNAc increased UDP-GlcNAc levels in *C. elegans* and also extended lifespan (38). Therefore, we asked whether GlcNAc salvage can rescue DTC phase 2 defects. When fed *AL* at 20^o^C, the *ngat-1(ev821)* defect was rescued by GlcNAc supplementation from 71% to 6%, and *ngat-1(ev840)* from 59% to 14% (Fig. 5*A*, Table S3). GALE-1 converts UDP-GlcNAc to UDP-GalNAc, a reversible reaction that balances the donor substrates for multiple pathways, including N-glycosylation and chondroitin biosynthesis (42) (Fig. 1*D*). Indeed, the *mgat-1* triple mutant was also rescued from 55% to 12% with added GlcNAc, presumably by increasing UDP-GalNAc flux to chondroitin synthesis. GlcNAc feeding was used in these experiments as the *gfat-1* and *ngat-1* genes are very close on chromosome II, making recombination infrequent.

**Figure 6:**
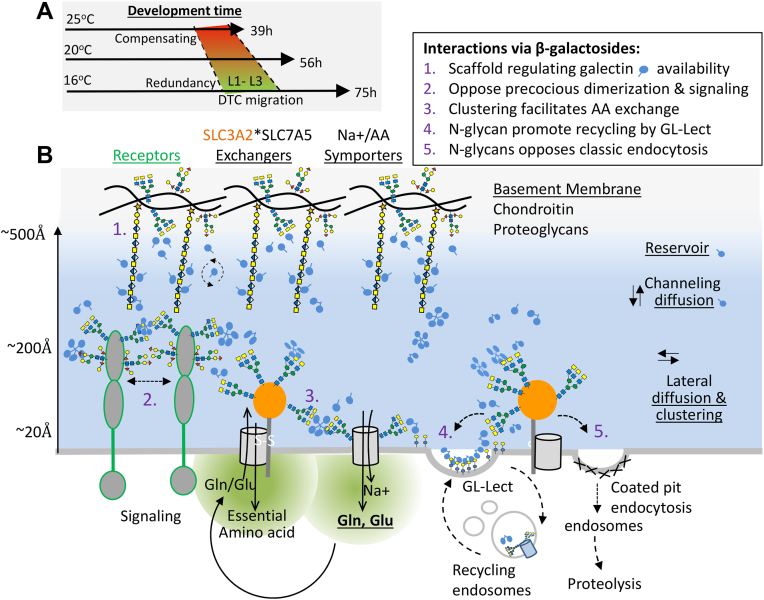
**Proposed scheme of β-galactoside bioequivalence and complementary redundancy.***A*, rates of growth and development from zygote to adult increases with temperature. Cell division and enzyme reaction increase according to the Arrhenius equation in a range that preserved fertility (78). The dotted line represents the window of L1-L3 when DTCs migrate to form the gonad. Golgi enzymes required for completion of complex-type N-glycans or chondroitin elongation support DTC phase 2 migration with apparent redundancy at optimal conditions, and compensation for one-another at stressful temperatures, backed up by the HBP. *B*, chondroitin displays β-galactoside binding sites as repeating disaccharide in linear chains. β-galactosides (LacdiNAc) are on a subset of branched complex-type N-glycans found on transmembrane glycoproteins. Galectins bind monovalent ligands with low affinity, but display increased density-dependent avidity with rapid on/off rates at the cell surface, which regulates focal adhesion turnover, surface retention of growth factor receptors, passage through recycling endosomes, and clustering of amino acid exchangers in mammalian cells (43, 45, 57). As depicted here, the chondroitin chains in BM may regulate the diffusion rate and availability of galectins at the cell surface. Complex-type N-glycans and paucimannose depicted as present on the hybrid chondroitin proteoglycan MIG-6, and on integrins (PAT-3, INA-1) and growth factor receptors (*e.g.*, UNC-5), and solute transporters SLC7A5 or PEPT-1 at the cell surface.

Next, we asked whether the gain-of-function mutants, *mig-22(k185gf****)*** and *gfat-1(dh468gf),* could rescue DTC phase 2 mutant defects. The *mig-22(k185gf)* mutation was isolated in a screen for suppressors of the DTC phase 2 defect in the *mig-17(k174)* null mutant and shown to increase chondroitin levels ∼2 fold and extend lifespan (37). *mig-22(gf****)*** or S/R partially rescued the phase 2 defect in *mig-17(k174)*, suggesting that S/R increases chondroitin synthesis beyond wild-type levels, which may, in turn, recruit one of the other seven ADAM paralogues in *C. elegans* (Fig. 5*B*, Table S4). Importantly, *mig-22(gf****)*** also strongly rescued the *ngat-1* mutant, presumably increasing chondroitin chain length that compensates for the missing LacdiNAc termini on the N-glycans of glycoproteins. *gfat-1(gf)* partially rescued *mig-17(k174)* although less effectively than S/R. In contrast, *gfat-1(gf)* rescued *mig-22(k141)* more effectively than S/R, suggesting that S/R increases HBP flux to UDP-GalNAc, but less efficiently than constitutive expression of *gfat-1(gf)* (Fig. 5*B*). Furthermore, the *ngat-1(ev840);mig-22(k141)* double mutant was completely insensitive to GlcNAc supplementation (95% with, 96% without GlcNAc) (Fig. 5*A*, Table S3), consistent with a requirement for increased UDP-GlcNAc and its conversion to UDP-GalNAc for the synthesis of chondroitin to rescue the *ngat-1* mutant defect. GlcNAc supplementation, *gfat-1(gf)* or *mig-22(gf****)*** expression are expected to increase chondroitin and make a denser web of β-galactosides predicted to increase local galectin concentrations and their effects on membrane receptors (43, 44), and nutrient transporters (45, 46) (see Fig. 6, *A* and *B*).

## Discussion

We were intrigued by the unusual properties of the DTC phase 2 defect in the *mgat-1* triple and *ngat-1* mutants, and genetic experiments that suggested a common pathway. Notably, DTC phase 2 defects in both mutants displayed a higher sensitivity to heat in the posterior than anterior. We infer that NGAT-1 catalyzes a late-stage LacdiNAc modification of complex-type N-glycans in the Golgi, and therefore its absence is unlikely to impair glycoprotein folding in the ER but is required in another capacity. The rescue of *ngat-1(lf)* by *mig-22(gf)* or GlcNAc feeding is not expected to restore the LacdiNAc on N-glycans but does rescue *ngat-1(lf)* phase 2 defects, suggesting that an increase in chondroitin compensates for the loss of LacdiNAc, perhaps on a thermolabile hybrid proteoglycan (*e.g.* MIG-6), bearing both types of glycans (47). By extension, the strong rescue of *ngat-1(null)* phase 2 defects by S/R likely occurs by a compensating increase in β-galactosides in the form of chondroitin, as phase 2 defects in *ngat-1;mig-22* double mutant are not rescued by S/R. Taken together with the gain-of-function data, the S/R rescue of *ngat-1(lf)* requires enhanced MIG-22 function and presumably up-regulation of HBP to levels higher than in *AL-fed* conditions. S/R rescue of the hypomorphic *mig-22(lf)* is also likely due to increased HBP flux to chondroitin synthesis and/or LacdiNAc synthesis by endogenous NGAT-1.

The temperature-sensitive period for NGAT-1 and MGAT-1 function was determined to be in early L1 - nearly three larval stages prior to the affected phase 2 migration (22). Wild-type forms of *ngat-1*, *mig-22* and *mig-17* rescue their respective mutants when expressed in the DTCs or in cell types that contact the DTCs during their migration, notably the epidermis for *mig-22* (11, 20) and body wall muscle for *ngat-1* and *mig-17* mutants (22). Thus, rescue is not restricted to cell type, but presumably by the concentration of extracellular β-galactosides made available three larval stages prior to the phase 2 turn. The MIG-17 ADAM protease is normally secreted from the body wall muscle cells then localizes to the gonad BM where DTC phase 2 occurs (11, 17). In *mig-23(k180)* and *gale-1(pv18)* mutants, MIG-17 is secreted but fails to accumulate in the gonadal BM. MIG-23 and GALE-1 expression in the muscle cells are both required for Golgi remodeling of N-glycans on MIG-17 and its subsequent localization (11, 17), consistent with the suggestion that other glycoproteins may have similar requirements (Figs. 3*G* and 6, A and B).

Chondroitin mutants, *mig-22* and *sqv-5* are highly penetrant at 16 °C and not very temperature sensitive, whereas *ngat1* and *mgat-1* have low penetrance at 16 °C and defects increase steeply with rising temperature, suggesting that heat sensitivity resides within the N-glycan structures. The expected depletion of LacdiNAc on complex-type N-glycans in *ngat-1* leaves behind only α- and β-galactosides on paucimannose (Galα/β1-4Fuc and Galα/β1-4Man) (27, 48), which may be marginally sufficient for phase 2 migration at low temperature, but not structurally suited for the molecular dynamics of galectin interactions at higher temperatures. Paucimannose has fewer β-galactosides and more α-glycosidic linkages, which may alter torsion angles at the glycosidic linkage (ϕ′ and ψ') and narrow conformational space (49, 50). Paucimannose has more substitutions at the chitobiose linkage to the protein, also reducing flexibility and galectin binding at higher temperatures. Biophysical analysis of galectin-glycan interactions has revealed that the change in conformational entropy (ΔS) is comparable to that of binding enthalpy (ΔH), thus consistent with weak binding (51), and rapid exchange (on/off rates) in a multivalent glycoconjugate network (*e.g.* galectin lattice) (52, 53). In wild-type *C. elegans*, complex-type N-glycans with β-linked branches and β-LacdiNAc can sample more microstates that protect phase 2 fidelity at higher temperatures. Indeed, rates of *C. elegans* development increase up to 2-fold with rising temperatures, which adds fragility to developmental processes such as axon migrations (54) (Fig. 6*A*). Moreover, paucimannose glycans have been purged by natural selection leading to endothermic vertebrates (mammals and birds), leaving complex-type N-glycans with higher levels of β-galactoside, presumably structures appropriate for life at higher temperatures.

As a model of cooperation, chondroitin chains may span the distance between BM and cell surface, thereby regulating galectin availability to transmembrane glycoproteins required for DTC phase 2 migration (Fig. 6*B*). Galectins can reduce the adhesive resistance of cells to substratum and promote α/β-integrin turnover as required for DTC motility (13). The migration rate of mammalian cells is highest at intermediate galectin-3 and β-integrin concentrations, as well as intermediate matrix stiffness (55, 56, 57). Titration of these essential components reveals biphasic responses, characteristic of multivalent systems (55, 57). The intrinsically disordered regions (IDR) in mammalian galectin-3 mediate self-association, and the galectin carbohydrate recognition domain (CRD) crosslinks membrane glycoproteins, impacting endocytosis, surface levels of receptor kinase (43, 44) and nutrient transporter (45, 46).

As a precedent for interaction between a galectin and glycoconjugates required for DTC migration, MIG-6 is reported to be a major binding partner for LEC-1, a galectin with two CRDs, and *lec-1* mutants display increased sensitivity to oxidative stress (58, 59). Feeding Galβ1-4Fuc-functionalized dendrimers to wild-type *C. elegans* protects against oxidative stress (60), perhaps by acting like a multivalent antagonist for the exchange of LEC-1 with glycoproteins and proteoglycans at the cell surface.

S/R is similar to periodic fasting studies in mammals, which have prolonged benefits to health and longevity (61, 62, 63), perhaps due in part to HBP and glycoconjugate pathways. In cultured mammalian cells, mutations in SLC3A2/SLC7A5, an essential amino acid exchanger, reduce mTOR signaling (64) as does starvation (65). Deletion of SLC3A2, the N-glycosylated adaptor subunit, reduces amino acid levels, leading to expression of stress-related *xbp1, GADD34 and CHOP,* and increases UDP-GlcNAc 2 to 3 fold (45), levels comparable to *gfat(gf)* expression (38). Our results suggest that S/R induces a similar increase in HBP activity required to rescue the *ngat-1* and *mig-22* phase 2 defects. The SLC3A2/SLC7A5 result is conceptually similar to the *pept-1* mutation in *C. elegans,* which reduces amino acid uptake, TOR/RSKS-1 signaling and translation. In preliminary experiments, DTC phase 2 defects at 20 °C were reduced from 44% in *ngat-1(ev840)* to 10% in *ngat-1(ev840);pept-1(lg601)* and to 16% in *ngat-1(ev840);rsks-1(ok1255)* double mutants. The normal L1 - L3 stage of development may involve ER stress that increases further by mutations in glycan synthesis (17, 66). The *pept-1* and *rsks-1* mutations are expected to slow translation and restore equilibrium with glycosylation pathways, thereby increasing the probability of normal phase 2 migration (67).

*mig-22(k185gf)* and *gfat-1(dh468gf)* mutations and GlcNAc supplementation have been shown to promote longevity and health span in *C. elegans* (37, 38), and downstream ECM glycoconjugates are associated with pathways of aging (68). Chondroitin supplements appear to extend lifespan (69), as does GlcNAc supplements in humans (70) and *C. elegans* (71). Conversely, glial scar formation at sites of CNS injury in adults (but not youth) has excessive deposition of chondroitin and ECM proteins that impair axon regeneration (72), observations consistent with biphasic responses to substratum composition, as noted above (55, 57). Our model for chondroitin and N-glycan interaction is speculative, but hopefully it will stimulate discussion and ultimately be tested by other groups, perhaps in models of aging.

## Experimental procedures

### Nematode culture

Standard procedures were used for the culture, maintenance and genetic analysis of *C. elegans* (73, 74) except that the standard amount of peptone was doubled in Nematode Growth Media (NGM) plates. The N2 Bristol strain was used as the standard wild-type strain. N2-derived *him-5* mutant males were used to start most crosses for making double and triple mutants. Mutations used here all cause loss-of-function *(lf)* defects, while those causing a gain-of-function are designated as *(gf)* (Table 1).

**Table 1:** Mutations used in this study

Mutation	Linkage Group	Mutation Type^a^	Predicted function	Activities
ngat-1 (ev840)	II	Large Deletion	Null	GalNAc-transferase
ngat-1 (ev821)	II	Deletion	Slight Antagonist	
ngat-1 (ev823)	II	N- terminal Deletion	Hypomorph	
mig-22 (k141)	III	G to E substitution	Hypomorph	chondroitin synthetase
mig-22 (k175gf)	III	Substitution	Gain-of-function	
sqv-5 (k172)	I	G to E Substitution	Hypomorph	chondroitin synthetase
mig-17 (k174)	V	Large deletion	Null	ADAM protease 9 N-glycan sites
gly-12 (id47)	X	Large deletion	Null	MGAT-1 transferase
gly-13 (ok712)	X	Large deletion	Null	MGAT-1 transferase
gly-14 (id48)	III	Large Deletion	Null	MGAT-1 transferase
mig-23 (k180)	X	Transposon insertion	Null	NDP-phosphodiesterase
gfat-1 (dh468gf)	II	Substitution	Gain-of-function	glutamine fructose-6-P aminotransferase
mig-6 (k177)	V	K to D substitution	Hypomorph	chondroitin proteoglycan with 18 N-glycan sites
cogc-1 (k179)	I	Transposon insertion	Null	Golgi complex trafficking
bre-4 (ok3167)	I	Deletion	Null	intestinal GalNAc-transferase

a Evidence from Wormbase.com.

Strains not isolated in our laboratories were obtained from K. Nishiwaki (Kwansei Gakuin University), A. Antebi (Max Planck Institute for Biology of Ageing), or the *C. elegans* Genetics Center, courtesy of T. Stiernagle (University of Minnesota). Most mutations were assayed by PCR.

### Phenotypic characterization of DTC phase 2 migration defects

#### Anatomical characterization by microscopy

DTC phase 2 mis-migrations often cause proximal and distal portions of the translucent gonad arm to lie side by side, pushing the dark intestine dorsally, thereby creating a ventral clear patch (VCP) visible by stereomicroscopy and useful for genetic studies as in Figure 2. Not all phase 2 defects are detected as VCPs using stereomicroscopy. Therefore, defects were also scored by differential interference contrast microscopy in L4 stage and young adult animals, a stage when zygotes are not yet present in the uterus to distort the shape of the gonad arms and increase their girth as observed in older mutant animals. Worms were placed on a 4% agarose pad and immobilized with 5 to 10 μl of 25 μM sodium azide. Differential interference contrast microscopy of gonad arms was performed using a Leica DMRA2 microscope, and images were captured using a Hamamatsu ORCA-ER digital camera. Criteria for a normal phase 2 trajectory were as follows: Normal phase 1 along the ventral BWMs is indicated by a linear trajectory of the gonad arm/tube along the ventral side (Fig. 2). This is followed by the phase 2 turn toward the dorsal side with no overlap between the first and second phase portions of the gonad arm tubes. The extent of overlap in mutants varies from half the length of the gonad arm to a barely discernible overlap (see Fig. 2*C*). If the turn is approximately 90° with no overlap between the proximal gonad arm formed during phase 1 and phase 2, and where the phase 2 portion extends directly to the dorsal BWMs with no obvious meandering, it is scored as wild type. Deviations from these criteria are scored as mutant. It is worth noting that a small minority of unguided migrations could, by chance, occur in the wild-type direction (maximum defect score is ∼90%).

#### Genetic analyses

The *ngat-1* mutants were backcrossed to N2 (wild type) at least two times before characterization. The *gly-14; gly-12 gly-13* triple mutant was originally isolated and made available by Drs. Harry Schachter and Wendy Johnston for characterization (25). For comparisons of different mutants grown at the same temperature, the animals were grown in parallel, *i.e.*, in the same incubator space, to correct for possible differences in incubator temperature, which could vary by 0.5 °C.

Starvation was induced using purified embryos, including those developing *in utero* prepared by treating gravid adults and ex utero embryos for 15 min at the given temperature with alkaline hypochlorite (0.36 N potassium hydroxide plus 20% hypochlorite) before washing with sterile water. The alkaline hypochlorite dissolves the adult body, leaving viable developing embryos. The egg/embryo pellet was then placed on NGM pates lacking *E. coli* and allowed to hatch. This blocks development in the L1 stage. Approximately one or 2 days after the plating, the L1 larvae were refed *E. coli*, which induced further development, then the animals were scored as L4S and young adults. One or 2 days of starvation before refeeding did not cause significant differences in penetrance of defects (two experiments each for *ngat-1, mig-17, mig-22; p* > 0.3).

Differences in penetrance from one experiment to another were potentially minimized by growing mutant animals at the indicated temperature without starvation from conception to phenotypic analysis. However, even though the mothers of these animals had been shifted as larvae to the growth temperature of their progeny, the larval stage timing of that shift varied and was not noted, leaving open the possibility of temperature-dependent maternal or epigenetic effects that could cause variability in the penetrance of their progeny. This may account for the extreme variability in penetrance in the anterior, but not the posterior DTC in *mig-22(k141)* animals (Table S1*B*).

#### CRISPR Cas9 gene editing

To derive the *ngat-1(ev840)* null allele CRISPR guide RNA was designed to target the sequence TAGCATTACATAGATTAGTG within the first exon of the *ngat-1* gene. Plasmid pDD162 (75) expressing Cas9, the guide RNA and a visible co-injection marker were injected into the gonads of N2 worms and lines expressing the marker were recovered. Deletions and insertions in exon one were identified by polyacrylamide electrophoresis of PCR products and DNA sequence analysis. Standard molecular biology methods (76) were used unless otherwise noted.

#### GlcNAc feeding

A 0.5 M solution of GlcNAc (Sigma chemicals) in pH 6.0 potassium phosphate buffered water (phosphate concentration equivalent to that present in NGM plates used to grow *C. elegans*) was made, and 0.1 ml was added to *E. coli*-seeded NGM plates and allowed to soak in for at least 3 h before adding developing eggs which had been purified by treatment of gravid adults for 15 min with alkaline hypochlorite before washing several times with sterile water.

#### Statistical analysis

In each experiment, animals were scored as normal or phase 2 DTC defects, and the fraction of defects was recorded (see Tables S1–S4). Independent experiments, from 3 to 10, were done to characterize single mutants, and four or five for double mutants at each temperature (16 °C, 20 °C, and 25 °C, Table S1,S2). The data are predicted to follow a normal distribution, apart from anterior DTC migration defects in *mig-22(k141)* mutants at 25^o^C where animals appeared sick, compared to 20 °C which was used for subsequent experiments with *mig-22(k141)* (Table S1*B*). The fraction of normal DTC migration and defects from repeat experiments were summed, the 95% confidence intervals of proportions and significance of differences between experimental groups were calculated using Vassarstats.net (77). A standard two-tailed comparison of two proportions *p* < 0.05 was taken as a significant difference.

## Data availability

DTC defects were scored and recorded in the Culotti lab, see Tables S1–S4.

## Supporting information

This article contains supporting information.

## Conflict of interest

The authors declare that they have no conflicts of interest with the contents of this article.
