# Supplementary material for: Distal tip cell migration mutants of Caenorhabditis elegans are rescued by bioequivalent outputs from chondroitin and N-glycosylation pathways
Source: J Biol Chem. 2025 Nov 4;301(12):110895. doi: 10.1016/j.jbc.2025.110895 (PMC12721165; doi:10.1016/j.jbc.2025.110895)
Supplement: Table S1 [file mmc1.docx]

| **Table S1A: Penetrance of DTC phase 2 migration defects in glycosylation and glycoprotein defective mutants** | | | | | | | | | | | | | | | |
| --- | --- | --- | --- | --- | --- | --- | --- | --- | --- | --- | --- | --- | --- | --- | --- |
|  | | | **CONTINUOUSLY WELL-FED - *ad libitum (AL*)^1^** | | | | | | **STARVED AND RE-FED (S/R)^2^** | | | | | |  |
|  | | | **Anterior DTC** | | | **Posterior DTC** | | | **Anterior DTC** | | | **Posterior DTC** | | |  |
| **Exp’t#^3^** | **Strain^4^** | **Temp^5^** | **mutant/**  **total** | **%** | **95% CI^6^** | **mutant/**  **total** | **%** | **95%C.I.^6^** | **mutant/**  **total** | **%** | **95%C.I.^6^** | **mutant/**  **total** | **%** | **95%C.I^6^** | **hours**  **starved** |
| **TOTAL** | ***N2*** | **25^o^C** | **0/306** | **0%** | **0-2%** | **6/326** | **2%** | **1-4%** | **0/85** | **0%** | **0-5%** | **1/85** | **1%** | **0-7%** | **41** |
| **6/11/20** | ***him-5*** | **25^o^C** | **0/45** | **0%** | **0-10%** | **1/52** | **2%** | **0.1-5%** |  |  |  |  |  |  |  |
|  |  |  |  |  |  |  |  |  |  |  |  |  |  |  |  |
| **7/7/20** | ***mgat-1* triple** | **16^o^C** | **1/91** | **1%** | **0.1-7%** | **19/91** | **21%** | **13-31%** |  |  |  |  |  |  |  |
| **1/4/23** | ***mgat-1* triple** | **20^o^C** | **1/51** | **2%** | **0.3-10%** | **24/53** | **45%** | **33-59%** |  |  |  |  |  |  |  |
| **TOTAL** | ***mgat-1* triple** | **25^o^C** | **6/310** | **2%** | **1-4%** | **173/323** | **54%** | **48-59%** | **1/114** | **1%** | **0.1-6%** | **8/114** | **7%** | **3-14%** | **49** |
|  |  |  |  |  |  |  |  |  |  |  |  |  |  |  |  |
| **TOTAL** | ***ngat-1(ev821)*** | **16^o^C** | **3/157** | **2%** | **0.5-6%** | **13/186** | **7%** | **4-12%%** |  |  |  |  |  |  |  |
| **TOTAL** | ***ngat-1(ev821)*** | **20^o^C** | **14/250** | **6%** | **3-9%** | **116/251** | **48%** | **40-55%** | **1/84** | **1%** | **0.1-7%** | **2/84** | **2%** | **1-9%** | **48** |
| **TOTAL** | ***ngat-1(ev821)*** | **25^o^C** | **14/416** | **3%** | **2-6%** | **310/439** | **71%** | **67-79%** | **2/113** | **2%** | **0.3-7%** | **15/118** | **13%** | **8-20%** | **Dauer** |
| **TOTAL** | ***ngat-1(ev821)*** | **25^o^C** |  |  |  |  |  |  | **1/98** | **1%** | **0.1-6%** | **17/97** | **18%** | **11-27%** | **52** |
|  |  |  |  |  |  |  |  |  |  |  |  |  |  |  |  |
| **7/11/18** | ***ngat-1(ev840)*** | **16^o^C** | **0/76** | **0%** | **0-6%** | **10/77** | **13%** | **7-23%** |  |  |  |  |  |  |  |
| **TOTAL** | ***ngat-1(ev840)*** | **20^o^C** | **1/342** | **0.3%** | **0-1%** | **241/551** | **44%** | **40-48%** | **2/155** | **1%** | **0.2-5%** | **4/154** | **3%** | **1-7%** | **48-50** |
| **TOTAL** | ***ngat-1(ev840)*** | **25^o^C** | **6/491** | **1%** | **0.5-3%** | **296/501** | **59%** | **55-63%** | **1/81** | **1%** | **0.1-8%** | **5/86** | **6%** | **2-18%** | **49** |
|  |  |  |  |  |  |  |  |  |  |  |  |  |  |  |  |
| **TOTAL** | ***mig-17(k174)*** | **16^o^C** | **18/188** | **10%** | **6-15%** | **71/183** | **39%** | **32-46%** |  |  |  |  |  |  |  |
| **TOTAL** | ***mig-17(k174)*** | **20^o^C** | **73/447** | **16%** |  | **192/464** | **41%** |  | **15/203** | **7%** | **4-12%** | **29/205** | **14%** | **10-20%** | **45&49** |
| **TOTAL** | ***mig-17(k174)*** | **25^o^C** | **77/385** | **20%** | **16-24%** | **267/390** | **68%** | **64-73%** | **3/57** | **5%** | **1-16%** | **5/57** | **9%** | **3-20%** | **25** |
|  |  |  |  |  |  |  |  |  |  |  |  |  |  |  |  |
| **TOTAL** | ***mig-22(k141)*** | **16^o^C** | **49/193** | **25%** | **20-32%** | **123/195** | **63%** | **56-70%** | **9/117** | **8%** | **4-15%** | **20/114** | **17%** | **11-26%** | **52** |
| **TOTAL** | ***mig-22(k141)*** | **20^o^C** | **122/313** | **39%** | **34-44%** | **219/304** | **72%** | **67-77%** | **26/176** | **15%** | **10-21%** | **58/173** | **34%** | **27-41%** | **48&51** |
| **4/11/19** | ***mig-22(k141)*** | **20^o^C** |  |  |  |  |  |  | **0/83** | **0%** | **0.1-6%** | **2/84** | **2%** | **0-9%** | **Dauer** |
| **8/9/17** | ***mig-22(k141)*** | **23^o^C** |  |  |  |  |  |  | **5/66** | **8%** | **3-17%** | **18/67** | **27%** | **17-39%** | **72** |
| **TOTAL** | ***mig-22(k141)*** | **25^o^C** | **232/483** | **48%** | **44-53%** | **378/481** | **79%** | **75-82%** | **67/91** | **74%** | **63-82%** | **58/92** | **63%** | **52-73%** | **52** |
|  |  |  |  |  |  |  |  |  |  |  |  |  |  |  |  |
| **TOTAL** | ***sqv-5(k172)*** | **16^o^C** | **231/271** | **85%** | **80-89%** | **186/261** | **71%** | **65-77%** |  |  |  |  |  |  |  |
| **TOTAL** | ***sqv-5(k172)*** | **20^o^C** | **298/334** | **89%** | **85-92%** | **240/325** | **74%** | **69-78%** | **41/58** | **71%** | **57-82%** | **32/56** | **57%** | **43-70%** | **48** |
| **TOTAL** | ***sqv-5(k172)*** | **25^o^C** | **342/362** | **94%** | **91-97%** | **332/368** | **90%** | **87-93%** | **75/91** | **82%** | **73-89%** | **61/87** | **70%** | **59-79%** | **55** |
|  |  |  |  |  |  |  |  |  |  |  |  |  |  |  |  |
| **5/27/19** | ***mig-23(k180)*** | **16^o^C** | **45/81** | **56%** | **44-66%** | **79/89** | **89%** | **80-94** |  |  |  |  |  |  |  |
| **2/2/22** | ***mig-23(k180)*** | **20^o^C** | **31/51** |  |  | **43/52** | **83%** | **70-91%** |  |  |  |  |  |  |  |
| **TOTAL** | ***mig-23(k180)*** | **25^o^C** | **217/260** | **83%** | **78-87%** | **224/263** | **85%** | **80-89%** | **149/189** | **79%** | **72-84%** | **175/196** | **89%** | **84-93%** | **50&50** |
|  |  |  |  |  |  |  |  |  |  |  |  |  |  |  |  |
| **TOTAL** | ***mig-6(k177)*** | **16^o^C** | **133/313** | **82%** | **75-87%** | **274/330** | **83%** | **79-87%** |  |  |  |  |  |  |  |
| **1/5/18** | ***mig-6(k177)*** | **20^o^C** | **62/82** | **76%** | **65-84%** | **72/92** | **78%** | **68-86%** | **57/79** | **72%** | **61-81%** | **62/102** | **61%** | **51-70%** | **45** |
| **TOTAL** | ***mig-6(k177)*** | **23 ^o^C** | **131/172** | **76%** | **69-82%** | **147/181** | **81%** | **74-86%** | **43/64** | **67%** | **54-78** | **46/68** | **68%** | **55-78%** | **52** |
| **TOTAL** | ***mig-6(k177)*** | **25^o^C** | **143/155** | **92%** | **87-96%** | **139/164** | **85%** | **78-90%** |  |  |  |  |  |  |  |
|  |  |  |  |  |  |  |  |  |  |  |  |  |  |  |  |
| **TOTAL** | ***cogc-1(k179)*** | **16^o^C** | **63/86** | **79%** | **69-85%** | **75/87** | **86%** | **77-92%** |  |  |  |  |  |  |  |
| **9/26/22** | ***cogc-1(k179)*** | **20^o^C** | **14/66** | **21%** | **13-33%** | **49/62** | **79%** | **66-88** |  |  |  |  |  |  |  |
| **TOTAL** | ***cogc-1(k179)*** | **25^o^C** | **31/345** | **9%** | **6-13%** | **285/345** | **83%** | **78-86** | **10/270** | **4%** | **2-7%** | **130/259** | **50%** | **44-56%** | **48&56** |

**Details of data contributing to Summary Table S1A can be found in Table S1B. ^1^ ‘Continuously well-fed’ means fed with abundant E. coli food source for > 1 generation at the temperature indicated. ^2^ ‘Starved and re-fed’ means embryos free of *E. coli* food source were prepared by treating animals for 15-20 minutes at the growth temperature with mold killer (24% Chlorox bleach, in 03N KOH) to kill everything except embryos encased in resistant eggshells. In some experiments, animals were starved to dauer before being re-fed - as indicated in the last column. ^3^ Each number represents one or more (=TOTAL) experiments. ^4^ In some double mutants, only the allele (in italics) may be listed without the gene name due to space constraints. ^5^ Temp refers to the growth temperature maintained for more than one full generation before beginning the experiment. ^6^ 95%C.I. is the 95% confidence intervals, calculated as suggested by {Newcombe, 1998 #199} (see Vassarstats.net).**



| **Table S1B: Penetrance of DTC phase 2 migration defects in glycosylation and glycoprotein defective mutants (raw data)** | | | | | | | | | | | | | | | |
| --- | --- | --- | --- | --- | --- | --- | --- | --- | --- | --- | --- | --- | --- | --- | --- |
|  | | | **CONTINUOUSLY WELL-FED1 (*AL*)** | | | | | | **STARVED AND RE-FED^2^ (S/R)** | | | | | |  |
|  | | | **Anterior DTC** | | | **Posterior DTC** | | | **Anterior DTC** | | | **Posterior DTC** | | |  |
| **Exp’t#^3^** | **Strain^4^** | **Temp^5^** | **mutant/**  **total** | **%** | **95%C.I.^6^** | **mutant/**  **total** | **%** | **95%C.I.^6^** | **mutant/**  **total** | **%** | **95%C.I.^6^** | **mutant/**  **total** | **%** | **95%C.I^6^** | **hours**  **starved** |
| **6/22/17** | ***N2*** | **25^o^C** | **0/107** | **0%** | **0.1-4%** | **4/115** | **3.5%** | **1-8%** |  |  |  |  |  |  |  |
| **6/23/17** | ***N2*** | **25^o^C** | **0/14** | **0%** | **0-27%** | **0/18** | **0%** | **0-22%** |  |  |  |  |  |  |  |
| **6/26/17** | ***N2*** | **25^o^C** | **0/74** | **0%** | **0-6%** | **1/82** | **1%** | **0.1-7%** |  |  |  |  |  |  |  |
| **6/16/20** | ***N2*** | **25^o^C** | **0/111** | **0%** | **0-4.3%** | **1/111** | **1%** | **0.1-6%** |  |  |  |  |  |  |  |
| **TOTAL** | ***N2*** | **25^o^C** | **0/306** | **0%** | **0-1.5%%** | **6/326** | **2%** | **0.7-4%** |  |  |  |  |  |  |  |
| **6/11/20** | ***him-5*** | **25^o^C** | **0/45** | **0%** | **0-10%** | **1/52** | **2%** | **0.1-5%** |  |  |  |  |  |  |  |
| **8/12/18** | ***N2*** | **25^o^C** |  |  |  |  |  |  | **0/85** | **0%** | **0-5%** | **1/85** | **1%** | **0-7%** | **41** |
|  |  |  |  |  |  |  |  |  |  |  |  |  |  |  |  |
| **7/7/20** | ***mgat-1* triple** | **16^o^C** | **1/91** | **1%** | **0.1-7%** | **19/91** | **21%** | **13-31%** |  |  |  |  |  |  |  |
| **1/4/23** | ***mgat-1* triple** | **20^o^C** | **1/51** | **2%** | **0.3-10%** | **24/53** | **45%** | **33-59%** |  |  |  |  |  |  |  |
| **3/29/17** | ***mgat-1* triple** | **25^o^C** | **3/65** | **5%** | **1.2-14%** | **29/69** | **43%** | **31-55%** |  |  |  |  |  |  |  |
| **3/31/17** | ***mgat-1* triple** | **25^o^C** | **2/58** | **4%** | **0.6-13%** | **29/65** | **45%** | **32-57%** |  |  |  |  |  |  |  |
| **4/4/17** | ***mgat-1* triple** | **25^o^C** | **1/65** | **2%** | **0.1-9%** | **37/65** | **57%** | **44-69%** |  |  |  |  |  |  |  |
| **7/6/20** | ***mgat-1* triple** | **25^o^C** | **0/122** | **0%** | **0-4%** | **78/124** | **62%** | **53-71%** |  |  |  |  |  |  |  |
| **TOTAL** | ***mgat-1* triple** | **25^o^C** | **6/310** | **2%** | **1-4%** | **173/323** | **54%** | **48-59%** |  |  |  |  |  |  |  |
| **3/29/17** | ***mgat-1* triple** | **25^o^C** |  |  |  |  |  |  | **2/73** | **3%** | **1-9%** | **17/78** | **22%** | **14-33%** | **7-10** |
| **4/4/17** | ***mgat-1* triple** | **25^o^C** |  |  |  |  |  |  | **1/75** | **1%** | **0.1-8%** | **16/79** | **20%** | **12-31%** | **4-8** |
| **7/12/20** | ***mgat-1* triple** | **25^o^C** |  |  |  |  |  |  | **1/114** | **1%** | **0.1-6%** | **8/114** | **7%** | **14%** | **49** |
|  |  |  |  |  |  |  |  |  |  |  |  |  |  |  |  |
| **9/27/23** | ***ngat-1(ev821)*** | **16^o^C** | **1/72** | **1%** | **0.1-89** | **8/78** | **10%** | **5-20%** |  |  |  |  |  |  |  |
| **8/28/23** | ***ngat-1(ev821)*** | **16^o^C** | **1/20** | **5%** | **0.3-27%** | **3/32** | **9%** | **2-26%** |  |  |  |  |  |  |  |
| **9/18/23** | ***ngat-1(ev821)*** | **16^o^C** | **1/65** | **2%** | **0.1-9%** | **2/76** | **3%** | **0.5-10%** |  |  |  |  |  |  |  |
| **TOTAL** | ***ngat-1(ev821)*** | **16^o^C** | **3/157** | **2%** | **0.5-6%** | **13/186** | **7%** | **4-12%** |  |  |  |  |  |  |  |
| **1/14/19** | ***ngat-1(ev821)*** | **20^o^C** | **2/70** | **3%** | **0.5-11%** | **30/70** | **43%** | **31-55%** |  |  |  |  |  |  |  |
| **3/15/19** | ***ngat-1(ev821)*** | **20^o^C** | **11/92** | **3%** | **1-9%** | **39/93** | **42%** | **32-53%** |  |  |  |  |  |  |  |
| **3/18/19** | ***ngat-1(ev821)*** | **20^o^C** | **1/88** | **1%** | **0.1-7%** | **47/88** | **53%** | **43-64%** |  |  |  |  |  |  |  |
| **TOTAL** | ***ngat-1(ev821)*** | **20^o^C** | **14/250** | **6%** | **3-9%** | **116/251** | **48%** | **40-55%** |  |  |  |  |  |  |  |
| **3/21/19** | ***ngat-1(ev821)*** | **20^o^C** |  |  |  |  |  |  | **1/84** | **1%** | **0.1-7%** | **2/84** | **2%** | **0.4-9%** | **48** |
| **2/6/17** | ***ngat-1(ev821)*** | **25^o^C** | **1/28** | **4%** | **0.2-20%** | **26/36** | **72%** | **55-85%** |  |  |  |  |  |  |  |
| **2/9/17** | ***ngat-1(ev821)*** | **25^o^C** | **5/36** | **14%** | **5-30%** | **25/38** | **66%** | **49-80%** |  |  |  |  |  |  |  |
| **3/15/17** | ***ngat-1(ev821)*** | **25^o^C** | **2/50** | **4%** | **1-15%** | **37/53** | **72%** | **57-83%** |  |  |  |  |  |  |  |
| **3/24/17** | ***ngat-1(ev821)*** | **25^o^C** | **3/44** | **7%** | **2-20%** | **33/46** | **73%** | **58-85%** |  |  |  |  |  |  |  |
| **5/25/17** | ***ngat-1(ev821)*** | **25^o^C** | **0/23** | **0%** | **0-18%** | **20/27** | **74%** | **53-88%** |  |  |  |  |  |  |  |
| **8/12/18** | ***ngat-1(ev821)*** | **25^o^C** | **1/46** | **2%** | **0.1-13%** | **30/48** | **63%** | **47-76%** |  |  |  |  |  |  |  |
| **5/16/18** | ***ngat-1(ev821)*** | **25^o^C** | **0/78** | **0%** | **0-6%** | **57/82** | **70%** | **58-79%** |  |  |  |  |  |  |  |
| **7/22/20** | ***ngat-1(ev821)*** | **25^o^C** | **2/111** | **2%** | **0.1-6%** | **82/109** | **75%** | **66-83%** |  |  |  |  |  |  |  |
| **TOTAL** | ***ngat-1(ev821)*** | **25^o^C** | **14/416** | **3%** | **2-6%** | **310/439** | **71%** | **67-75%** |  |  |  |  |  |  |  |
| **3/17/17** | ***ngat-1(ev821)*** | **25^o^C** |  |  |  |  |  |  | **2/62** | **3%** | **1-12%** | **25/65** | **36%** | **23-49%** | **4-8** |
| **7/24/20** | ***ngat-1(ev821)*** | **25^o^C** |  |  |  |  |  |  | **1/98** | **1%** | **0.1-6%** | **17/97** | **18%** | **11-27%** | **52** |
| **3/20/17** | ***ngat-1(ev821)*** | **25^o^C** |  |  |  |  |  |  | **2/77** | **3%** | **0.5-10%** | **14/82** | **17%** | **10-27%** | **Dauer** |
| **2/3/17** | ***ngat-1(ev821)*** | **25^o^C** |  |  |  |  |  |  | **0/36** | **0%** | **0-10%** | **1/36** | **3%** | **0.2-16%** | **Dauer** |
|  |  |  |  |  |  |  |  |  |  |  |  |  |  |  |  |
| **7/11/18** | ***ngat-1(ev840)*** | **16^o^C** | **0/76** | **0%** | **0-6%** | **10/77** | **13%** | **7-23%** |  |  |  |  |  |  |  |
| **1/25/19** | ***ngat-1(ev840)*** | **20^o^C** | **0/85** | **0%** | **0-5%** | **41/85** | **48%** | **37-59%** |  |  |  |  |  |  |  |
| **2/4/19** | ***ngat-1(ev840)*** | **20^o^C** | **N.D.** |  |  | **105/208** | **50%** | **43-57%** |  |  |  |  |  |  |  |
| **11/21/18** | ***ngat-1(ev840)*** | **20^o^C** | **0/65** | **0%** | **0-7%** | **26/66** | **39%** | **28-52%** |  |  |  |  |  |  |  |
| **3/14/19** | ***ngat-1(ev840)*** | **20^o^C** | **1/117** | **1%** | **0.4-5%** | **44/117** | **38%** | **29-47%** |  |  |  |  |  |  |  |
| **3/21/19** | ***ngat-1(ev840)*** | **20^o^C** | **0/75** | **0%** | **0-6%** | **25/75** | **33%** | **23-45%** |  |  |  |  |  |  |  |
| **TOTAL** | **ngat-1(ev840)** | **20^o^C** | **1/342** | **0.3%** | **0-2%** | **241/551** | **44%** | **40-48%** |  |  |  |  |  |  |  |
| **3/10/19** | ***ngat-1(ev840)*** | **20^o^C** |  |  |  |  |  |  | **2/83** | **2%** | **1-9%** | **2/83** | **2%** | **1-9%** | **43** |
| **3/21/19** | ***ngat-1(ev840)*** | **20^o^C** |  |  |  |  |  |  | **0/91** | **0%** | **0-5%** | **2/91** | **2%** | **1-8%** | **48** |
| **3/30/19** | ***ngat-1(ev840)*** | **20^o^C** |  |  |  |  |  |  | **2/64** | **3%** | **1-12%** | **2/63** | **3%** | **1-12%** | **50** |
| **TOTAL** | ***ngat-1(ev840)*** | **20^o^C** |  |  |  |  |  |  | **4/238** | **1.5%** | **0.5-5%** | **6/237** | **1%** | **0.5-6%** |  |
| **7/12/18** | ***ngat-1(ev840)*** | **25^o^C** | **2/88** | **2%** | **0.4-9%** | **51/98** | **56%** | **46-66%** |  |  |  |  |  |  |  |
| **8/17/18** | ***ngat-1(ev840)*** | **25^o^C** | **2/40** | **5%** | **0.9-18%** | **29/40** | **73%** | **56-85%** |  |  |  |  |  |  |  |
| **3/13/19** | ***ngat-1(ev840)*** | **25^o^C** | **0/102** | **0%** | **0-5%** | **51/102** | **50%** | **40-60%** |  |  |  |  |  |  |  |
| **4/3/19** | ***ngat-1(ev840)*** | **25^o^C** | **1/80** | **1%** | **1-8%** | **48/80** | **60%** | **48-71%** |  |  |  |  |  |  |  |
| **9/3/18** | ***ngat-1(ev840)*** | **25^o^C** | **0/55** | **0%** | **0-9%** | **36/56** | **64%** | **51-76%** |  |  |  |  |  |  |  |
| **11/21/20** | ***ngat-1(ev840)*** | **25^o^C** | **1/125** | **1%** | **0.1-5%** | **81/125** | **65%** | **56-73** |  |  |  |  |  |  |  |
| **TOTAL** | ***ngat-1(ev840)*** | **25^o^C** | **6/491** | **1%** | **0.5-3%** | **296/501** | **59%** | **55-63%** |  |  |  |  |  |  |  |
| **8/16/18** | ***ngat-1(ev840)*** | **25^o^C** |  |  |  |  |  |  | **2/82** | **2%** | **0.4-9%** | **6/85** | **7%** | **3-15%** | **27** |
| **4/4/19** | ***ngat-1(ev840)*** | **25^o^C** |  |  |  |  |  |  | **1/81** | **1%** | **0.1-8%** | **5/86** | **6%** | **1-12%** | **49** |
|  |  |  |  |  |  |  |  |  |  |  |  |  |  |  |  |
| **6/15/20** | ***mig-17(k174)*** | **16^o^C** | **12/107** | **11%** | **6-19%** | **42/104** | **40%** | **31-50%** |  |  |  |  |  |  |  |
| **6/24/20** | ***mig-17(k174)*** | **16^o^C** | **6/81** | **7%** | **3-16%** | **29/79** | **37%** | **26-48%** |  |  |  |  |  |  |  |
| **TOTAL** | ***mig-17(k174)*** | **16^o^C** | **18/188** | **10%** | **6-15%** | **71/183** | **39%** | **32-46%** |  |  |  |  |  |  |  |
| **7/17/19** | ***mig-17(k174)*** | **20^o^C** | **16/51** | **31%** | **20-45%** | **23/58** | **40%** | **27-53%** |  |  |  |  |  |  |  |
| **12/2/19** | ***mig-17(k174)*** | **20^o^C** | **11/44** | **25%** | **14-41%** | **21/49** | **43%** | **26-58%** |  |  |  |  |  |  |  |
| **12/4/19** | ***mig-17(k174)*** | **20^o^C** | **17/70** | **24%** | **16-33%** | **41/73** | **52%** | **42-61%** |  |  |  |  |  |  |  |
| **9/9/18** | ***mig-17(k174)*** | **20^o^C** | **2/78** | **3%** | **0.4-10%** | **34/81** | **42%** | **31-54%** |  |  |  |  |  |  |  |
| **7/4/20** | ***mig-17(k174)*** | **20^o^C** | **12/123** | **10%** | **5-17%** | **49/121** | **41%** | **32-50%** |  |  |  |  |  |  |  |
| **7/2/20** | ***mig-17(k174)*** | **20^o^C** | **15/81** | **19%** | **11-29%** | **24/82** | **29%** | **20-42%** |  |  |  |  |  |  |  |
| **TOTAL** | ***mig-17(k174)*** | **20^o^C** | **73/447** | **16%** | **13-20%** | **192/464** | **41%** | **37-46%** |  |  |  |  |  |  |  |
| **9/9/18** | ***mig-17(k174)*** | **20^o^C** |  |  |  |  |  |  | **3/57** | **5%** | **1-16%** | **5/57** | **9%** | **3-20%** | **25** |
| **01/18/20** | ***mig-17(k174)*** | **20^o^C** |  |  |  |  |  |  | **10/90** | **11%** | **6-20%** | **11/93** | **12%** | **6-21%** | **49** |
| **7/4/20** | ***mig-17(k174)*** | **20^o^C** |  |  |  |  |  |  | **5/113** | **4%** | **2-11%** | **18/112** | **16%** | **10-24%** | **45** |
| **TOTAL** | ***mig-17(k174)*** | **20^o^C** |  |  |  |  |  |  | **‘15/203** | **7%** | **4-12%** | **29/205** | **14%** | **10-20%** | **45&49** |
| **12/29/18** | ***mig-17(k174)*** | **25^o^C** | **16/47** | **34%** | **21-49%** | **40/49** | **82%** | **6-91%** |  |  |  |  |  |  |  |
| **7/16/19** | ***mig-17(k174)*** | **25^o^C** | **16/70** | **23%** | **15-34%** | **52/68** | **76%** | **65-85%** |  |  |  |  |  |  |  |
| **11/27/17** | ***mig-17(k174)*** | **25^o^C** | **5/35** | **14%** | **5-31%** | **21/37** | **57%** | **40-73%** |  |  |  |  |  |  |  |
| **12/8/18** | ***mig-17(k174)*** | **25^o^C** | **3/36** | **8%** | **2-24%** | **23/36** | **64%** | **46-79%** |  |  |  |  |  |  |  |
| **1/30/20** | ***mig-17(k174)*** | **25^o^C** | **26/90** | **29%** | **20-40%** | **68/97** | **70%** | **60-79%** |  |  |  |  |  |  |  |
| **6/13/20** | ***mig-17(k174)*** | **25^o^C** | **11/107** | **10%** | **5-18%** | **63/103** | **61%** | **51-70%** |  |  |  |  |  |  |  |
| **TOTAL** | ***mig-17(k174)*** | **25^o^C** | **77/385** | **20%** | **16-24%** | **267/390** | **68%** | **64-73%** |  |  |  |  |  |  |  |
| **9/09/18** | ***mig-17(k174)*** | **25^o^C** |  |  |  |  |  |  | **3/57** | **5%** | **1-16%** | **5/57** | **9%** | **3-20%** | **25** |
|  |  |  |  |  |  |  |  |  |  |  |  |  |  |  |  |
| **11/22/18** | ***mig-22(k141)*** | **16^o^C** | **18/29** | **62%** | **42-79%** | **16/29** | **55%** | **36-73** |  |  |  |  |  |  |  |
| **8/15/17** | ***mig-22(k141)*** | **16^o^C** | **5/27** | **19%** | **7-39%** | **29/35** | **83%** | **66-93%** |  |  |  |  |  |  |  |
| **10/27/18** | ***mig-22(k141)*** | **16^o^C** | **18/66** | **27%** | **17-40%** | **31/58** | **53%** | **40-66%** |  |  |  |  |  |  |  |
| **12/23/21** | ***mig-22(k141)*** | **16^o^C** | **8/71** | **11%** | **5-22%** | **47/73** | **64%** | **52-75%** |  |  |  |  |  |  |  |
| **TOTAL** | ***mig-22(k141)*** | **16^o^C** | **49/193** | **25%** | **20-32%** | **123/195** | **63%** | **56-70%** |  |  |  |  |  |  |  |
| **8/25/20** | ***mig-22(k141)*** | **16^o^C** |  |  |  |  |  |  | **9/113** | **8%** | **4-15%** | **20/114** | **17%** | **11-26%** | **52** |
| **12/24/18** | ***mig-22(k141)*** | **20^o^C** | **6/32** | **19%** | **8-37%** | **24/31** | **77%** | **58-90%** |  |  |  |  |  |  |  |
| **2/22/19** | ***mig-22(k141)*** | **20^o^C** | **17/51** | **33%** | **21-48%** | **34/51** | **65%** | **50-77%** |  |  |  |  |  |  |  |
| **8/2/18** | ***mig-22(k141)*** | **20^o^C** | **21/71** | **30%** | **20-42%** | **46/64** | **72%** | **59-82%** |  |  |  |  |  |  |  |
| **4/17/19** | ***mig-22(k141)*** | **20^o^C** | **58/102** | **46%** | **36-56%** | **70/97** | **72%** | **62-81%** |  |  |  |  |  |  |  |
| **12/9/22** | ***mig-22(k141)*** | **20^o^C** | **20/57** | **35%** | **23-49%** | **45/61** | **74%** | **61-84%** |  |  |  |  |  |  |  |
| **TOTAL** | ***mig-22(k141)*** | **20^o^C** | **122/313** | **39%** | **34-44%** | **219/304** | **72%** | **67-77%** |  |  |  |  |  |  |  |
| **4/11/19** | ***mig-22(k141)*** | **20^o^C** |  |  |  |  |  |  | **0/83** | **0%** | **0-6%** | **2/84** | **2%** | **0.4-9%** | **Dauer** |
| **12/23/22** | ***mig-22(k141)*** | **20^o^C** |  |  |  |  |  |  | **8/44** | **18%** | **10-32** | **18/46** | **36%** | **29-44** | **24** |
| **4/11/24** | ***mig-22(k141)*** | **20^o^C** |  |  |  |  |  |  | **9/77** | **12%** | **6-22%** | **23/80** | **29%** | **19-40%** | **48** |
| **4/19/19** | ***mig-22(k141)*** | **20^o^C** |  |  |  |  |  |  | **17/99** | **17%** | **11-26%** | **35/93** | **38%** | **28-48%** | **51** |
| **TOTAL** | ***mig-22(k141)*** | **20^o^C** |  |  |  |  |  |  | **26/176** | **15%** | **10-21%** | **58/173** | **34%** | **27-41%** | **48&51** |
| **8/9/17** | ***mig-22(k141)*** | **23^o^C** |  |  |  |  |  |  | **5/66** | **8%** | **3-17%** | **18/67** | **27%** | **17-39%** | **72 hrs** |
| **8/15/17** | ***mig-22(k141)*** | **25^o^C^7^** | **6/31** | **19%** | **8-38%** | **22/32** | **69%** | **50-83%** |  |  |  |  |  |  |  |
| **2/29/20** | ***mig-22(k141)*** | **25^o^C** | **29/34** | **85%** | **68-94%** | **31/34** | **91%** | **75-98** |  |  |  |  |  |  |  |
| **7/22/20** | ***mig-22(k141)*** | **25^o^C** | **27/98** | **28%** | **19-38%** | **61/93** | **66%** | **55-75%** |  |  |  |  |  |  |  |
| **7/24/20** | ***mig-22(k141)*** | **25^o^C** |  |  |  |  |  |  | **67/91** | **74%** | **63-82%** | **58/92** | **63%** | **52-73%** | **52** |
| **12/6/21** | ***mig-22(k141)*** | **25^o^C** | **4/13** | **31%** | **10-61%** | **11/14** | **79%** | **49-94%** |  |  |  |  |  |  |  |
| **12/9/21** | ***mig-22(k141)*** | **25^o^C** | **31/37** | **84%** | **67-93%** | **34/38** | **89%** | **74-97** |  |  |  |  |  |  |  |
| **12/17/21** | ***mig-22(k141)*** | **25^o^C** | **59/74** | **80%** | **68-88%** | **64/69** | **93%** | **83-97%** |  |  |  |  |  |  |  |
| **3/28/22** | ***mig-22(k141)*** | **25^o^C** | **23/29** | **79%** | **60-91%** | **25/30** | **83%** | **65-94%** |  |  |  |  |  |  |  |
| **4/12/22** | ***mig-22(k141)*** | **25^o^C** | **25/57** | **44%** | **31-58%** | **46/58** | **79%** | **66-8%8** |  |  |  |  |  |  |  |
| **4/14/22** | ***mig-22(k141)*** | **25^o^C** | **10/82** | **12%** | **6-22%** | **63/85** | **74%** | **63-83%** |  |  |  |  |  |  |  |
| **4/15/22** | ***mig-22(k141)*** | **25^o^C** | **18/28** | **64%** | **44-81%** | **21/28** | **75%** | **55-89%** |  |  |  |  |  |  |  |
| **TOTAL** | ***mig-22(k141)*** | **25^o^C** | **232/481** | **48%** | **44-53%** | **378/481** | **79%** | **75-82** | **67/91** | **74%** | **63-82%** | **58/92** | **63%** | **52-73%** | **52** |
|  |  |  |  |  |  |  |  |  |  |  |  |  |  |  |  |
| **12/13/19** | ***sqv-5(k172)*** | **16^o^C** | **93/106** | **88%** | **80-93** | **68/96** | **71%** | **61-80%** |  |  |  |  |  |  |  |
| **4/6/19** | ***sqv-5(k172)*** | **16^o^C** | **54/59** | **92%** | **81-97%** | **51/60** | **85%** | **73-93%** |  |  |  |  |  |  |  |
| **11/3/21** | ***sqv-5(k172)*** | **16^o^C** | **84/106** | **79%** | **70-86%** | **67/105** | **64%** | **54-72%** |  |  |  |  |  |  |  |
| **TOTAL** | ***sqv-5(k172)*** | **16^o^C** | **231/271** | **85%** | **80-89%** | **186/261** | **71%** | **65-77%** |  |  |  |  |  |  |  |
| **8/28/18** | ***sqv-5(k172)*** | **20^o^C** | **53/68** | **78%** | **66-87%** | **56/69** | **81%** | **70-89%** |  |  |  |  |  |  |  |
| **3/8/19** | ***sqv-5(k172)*** | **20^o^C** | **86/93** | **93%** | **85-97%** | **62/86** | **72%** | **61-77%** |  |  |  |  |  |  |  |
| **10/26/21** | ***sqv-5(k172)*** | **20^o^C** | **82/93** | **88%** | **79-94%** | **61/93** | **66%** | **55-75%** |  |  |  |  |  |  |  |
| **1/7/22** | ***sqv-5(k172)*** | **20^o^C** | **77/80** | **96%** | **89-99%** | **61/77** | **79%** | **68-87%** |  |  |  |  |  |  |  |
| **TOTAL** | ***sqv-5(k172)*** | **20^o^C** | **298/334** | **89%** | **85-92%** | **240/325** | **74%** | **69-78%** |  |  |  |  |  |  |  |
| **4/23/19** | ***sqv-5(k172)*** | **20^o^C** |  |  |  |  |  |  | **29/79** | **37%** | **26-48%** | **60/83** | **72%** | **61-81%** | **Dauer** |
| **8/28/18** | ***sqv-5(k172)*** | **20^o^C** |  |  |  |  |  |  | **69/92** | **75%** | **65-83%** | **68/102** | **67%** | **57-76%** | **25** |
| **2/22/19** | ***sqv-5(k172)*** | **20^o^C** |  |  |  |  |  |  | **41/58** | **71%** | **57-82%** | **32/56** | **57%** | **43-70%** | **48** |
| **4/2/19** | ***sqv-5(k172)*** | **25^o^C** | **78/82** | **95%** | **87-98%** | **79/82** | **96%** | **89-99%** |  |  |  |  |  |  |  |
| **12/13/19** | ***sqv-5(k172)*** | **25^o^C** | **20/22** | **91%** | **61-98%** | **25/26** | **96%** | **78-99%** |  |  |  |  |  |  |  |
| **01/09/20** | ***sqv-5(k172)*** | **25^o^C** | **80/83** | **96%** | **89-99%** | **72/88** | **82%** | **72-89%** |  |  |  |  |  |  |  |
| **01/16/20** | ***sqv-5(k172)*** | **25^o^C** | **79/84** | **94%** | **86-98%** | **74/80** | **93%** | **84-97%** |  |  |  |  |  |  |  |
| **05/8/18** | ***sqv-5(k172)*** | **25^o^C** | **23/29** | **79%** | **60-91%** | **25/30** | **83%** | **65-94%** |  |  |  |  |  |  |  |
| **12/30/21** | ***sqv-5(k172)*** | **25^o^C** | **62/62** | **100%** | **93-100%** | **57/62** | **92%** | **81-97%** |  |  |  |  |  |  |  |
| **TOTAL** | ***sqv-5(k172)*** | **25^o^C** | **342/362** | **94%** | **91-97%** | **332/368** | **90%** | **87-93%** |  |  |  |  |  |  |  |
| **4/23/19** | ***sqv-5(k172)*** | **25^o^C** |  |  |  |  |  |  | **30/70** | **43%** | **31-55%** | **57/78** | **73%** | **62-82%** | **Dauer** |
| **01/17/20** | ***sqv-5(k172)*** | **25^o^C** |  |  |  |  |  |  | **75/91** | **73%** | **82-89%** | **61/87** | **70%** | **59-79%** | **55** |
|  |  |  |  |  |  |  |  |  |  |  |  |  |  |  |  |
| **5/27/19** | ***mig-23(k180)*** | **16^o^C** | **45/81** | **56%** | **44-66%** | **79/89** | **89%** | **80-94%** |  |  |  |  |  |  |  |
| **5/27/19** | ***mig-23(k180)*** | **20^o^C** | **31/51** | **61%** | **46-74%** | **43/52** | **83%** | **69-91%** |  |  |  |  |  |  |  |
| **5/10/19** | ***mig-23(k180)*** | **25^o^C** | **91/96** | **95%** | **88-98%** | **87/99** | **88%** | **79-93%** |  |  |  |  |  |  |  |
| **8/10/17** | ***mig-23(k180)*** | **25^o^C** | **36/51** | **71%** | **56-82%** | **42/51** | **82%** | **67-91%** |  |  |  |  |  |  |  |
| **7/29/20** | ***mig-23(k180)*** | **25^o^C** | **90/113** | **80%** | **71-86%** | **95/113** | **84%** | **76-90%** |  |  |  |  |  |  |  |
| **TOTAL** | ***mig-23(k180)*** | **25^o^C** | **217/260** | **83%** | **78-87%** | **224/263** | **79%** | **74-84%** |  |  |  |  |  |  |  |
| **5/12/19** | ***mig-23(k180)*** | **25^o^C** |  |  |  |  |  |  | **82/88** | **93%** | **85-97%** | **76/88** | **86%** | **77-92%** | **50** |
| **8/10/17** | ***mig-23(k180)*** | **25^o^C** |  |  |  |  |  |  | **29/51** | **57%** | **42-70%** | **42/51** | **82%** | **69-91%** | **72** |
| **7/31/20** | ***mig-23(k180)*** | **25^o^C** |  |  |  |  |  |  | **67/101** | **65%** | **55-74%** | **99/108** | **92%** | **84-96%** | **50** |
|  |  |  |  |  |  |  |  |  |  |  |  |  |  |  |  |
| **12/27/17** | ***mig-6(k177)*** | **16^o^C** | **23/55** | **42%** | **29-56%** | **48/61** | **77%** | **66-88%** |  |  |  |  |  |  |  |
| **6/23/20** | ***mig-6(k177)*** | **16^o^C** | **40/97** | **42%** | **32-52%** | **89/105** | **85%** | **76-91%** |  |  |  |  |  |  |  |
| **9/15/21** | ***mig-6(k177)*** | **16^o^C** | **51/84** | **61%** | **49-71%** | **69/88** | **78%** | **68-86%** |  |  |  |  |  |  |  |
| **12/22/21** | ***mig-6(k177)*** | **16^o^C** | **19/77** | **25%** | **16-36%** | **68/76** | **89%** | **80-95%** |  |  |  |  |  |  |  |
| **TOTAL** | ***mig-6(k177)*** | **16^o^C** | **163/313** | **5%** | **46-57%** | **274/330** | **83%** | **78-87%** |  |  |  |  |  |  |  |
| **1/5/18** | ***mig-6(k177)*** | **20^o^C** | **62/82** | **76%** | **65-84%** | **72/92** | **78%** | **68-86%** |  |  |  |  |  |  |  |
| **1/5/18** | ***mig-6(k177)*** | **20^o^C** |  |  |  |  |  |  | **57/79** | **72%** | **61-81%** | **62/102** | **61%** | **51-70%** | **45** |
| **12/23/17** | ***mig-6(k177)*** | **23^o^C** | **49/65** | **75%** | **63-85%** | **58/68** | **86%** | **74-92%** |  |  |  |  |  |  |  |
| **12/19/17** | ***mig-6(k177)*** | **23 ^o^C** | **49/63** | **78%** | **65-87%** | **57/68** | **84%** | **72-91%** |  |  |  |  |  |  |  |
| **8/23/17** | ***mig-6(k177)*** | **23 ^o^C** | **33/44** | **75%** | **60-86%** | **32/45** | **71%** | **55-83%** |  |  |  |  |  |  |  |
| **TOTAL** | ***mig-6(k177)*** | **23 ^o^C** | **131/172** | **76%** | **69-82%** | **147/181** | **81%** | **74-86%** |  |  |  |  |  |  |  |
| **12/19/17** | ***mig-6(k177)*** | **23^o^C** |  |  |  |  |  |  | **43/64** | **67%** | **54-78** | **46/68** | **68%** | **55-78%** | **52** |
| **8/23/17** | ***mig-6(k177)*** | **23 ^o^C** |  |  |  |  |  |  | **22/38** | **58%** | **41-73%** | **31/40** | **78%** | **61-89%** | **24** |
| **6/24/20** | ***mig-6(k177)*** | **25^o^C** | **72/79** | **91%** | **82-96%** | **67/83** | **81%** | **70-88%** |  |  |  |  |  |  |  |
| **12/15/21** | ***mig-6(k177)*** | **25^o^C** | **71/76** | **93%** | **85-98%** | **72/81** | **89%** | **79-94%** |  |  |  |  |  |  |  |
| **TOTAL** | ***mig-6(k177)*** | **25^o^C** | **143/155** | **92%** | **87-96%** | **139/164** | **85%** | **78-90%** |  |  |  |  |  |  |  |
|  |  |  |  |  |  |  |  |  |  |  |  |  |  |  |  |
| **1/25/24** | ***cogc-1(k179)*** | **16^o^C** | **18/26** | **69%** | **48-85%** | **28/32** | **88%** | **70-96%** |  |  |  |  |  |  |  |
| **1/29/24** | ***cogc-1(k179)*** | **16^o^C** | **45/60** | **75%** | **62-85%** | **47/65** | **72%** | **60-82%** |  |  |  |  |  |  |  |
| **2/15/14** | ***cogc-1(k179)*** | **16^o^C** | **18/25** |  |  | **21/26** |  |  |  |  |  |  |  |  |  |
| **2/17/24** | ***cogc-1(k179)*** | **16^o^C** | **27/61** |  |  | **40/61** |  |  |  |  |  |  |  |  |  |
| **TOTAL** | ***cogc-1(k179)*** | **16^o^C** | **108/172** | **63%** | **55-70%** | **156/184** | **85%** | **79-99%** |  |  |  |  |  |  |  |
| **5/28/19** | ***cogc-1(k179)*** | **17^o^C** | **35/92** | **38%** | **28-49%** | **80/92** | **87%** | **78-92%** |  |  |  |  |  |  |  |
| **9/26/22** | ***cogc-1(k179)*** | **20^o^C** | **14/60** | **23%** | **14-36%** | **49/62** | **79%** | **66-88%** |  |  |  |  |  |  |  |
| **5/10/19** | ***cogc-1(k179)*** | **25^o^C** | **7/70** | **10%** | **4-20%** | **60/71** | **84%** | **74-92%** |  |  |  |  |  |  |  |
| **7/15/20** | ***cogc-1(k179)*** | **25^o^C** | **5/35** | **14%** | **5-31%** | **23/33** | **70%** | **51-84%** |  |  |  |  |  |  |  |
| **7/22/20** | ***cogc-1(k179)*** | **25^o^C** | **5/120** | **4%** | **2-10%** | **101/117** | **86%** | **78-92%** |  |  |  |  |  |  |  |
| **9/20/22** | ***cogc-1(k179)*** | **25^o^C** | **4/45** | **7%** | **3-17%** | **45/47** | **96%** | **86-99%** |  |  |  |  |  |  |  |
| **1/25/24** | ***cogc-1(k179)*** | **25^o^C** | **10/75** | **13%** | **7-24%** | **56/77** | **73%** | **61-82%** |  |  |  |  |  |  |  |
| **TOTAL** | ***cogc-1(k179)*** | **25^o^C** | **31/345** | **9%** | **6-13%** | **285/345** | **83%** | **78-86** |  |  |  |  |  |  |  |
| **5/12/19** | ***cogc-1(k179)*** | **25^o^C** |  |  |  |  |  |  | **1/87** | **1%** | **0.1-7%** | **32/85** | **38%** | **28-49%** | **50** |
| **7/18/20** | ***cogc-1(k179)*** | **25^o^C** |  |  |  |  |  |  | **8/76** | **11%** | **5-20%** | **41/70** | **59%** | **46-70%** | **48** |
| **7/24/20** | ***cogc-1(k179)*** | **25^o^C** |  |  |  |  |  |  | **1/107** | **1%** | **0.1-6%** | **57/104** | **55%** | **45-64%** | **56** |
| **TOTAL** | ***cogc-1(k179)*** | **25^o^C** |  |  |  |  |  |  | **9/183** | **5%** | **2-9%** | **130/259** | **50%** | **44-56%** | **48&56** |

**Table S1B footnotes as in Table S1A. ^7^ The reason for the large variation in mig-22(k141) mutants is unknown but could represent a form of epigenetics yet to be tested.**
