# Supplementary material for: Distal tip cell migration mutants of Caenorhabditis elegans are rescued by bioequivalent outputs from chondroitin and N-glycosylation pathways
Source: J Biol Chem. 2025 Nov 4;301(12):110895. doi: 10.1016/j.jbc.2025.110895 (PMC12721165; doi:10.1016/j.jbc.2025.110895)
Supplement: Table S2 [file mmc2.docx]

| **Table S2A: Penetrance of DTC phase 2 migration defects in double mutants of *ngat-1,* *mig-22(k141)* and *mig-17 (k174)*** | | | | | | | | | | | | | | | |
| --- | --- | --- | --- | --- | --- | --- | --- | --- | --- | --- | --- | --- | --- | --- | --- |
|  | | | **CONTINUOUSLY WELL-FED^1^ (*AL*)** | | | | | | **STARVED AND RE-FED^2^  (S/R)** | | | | | |  |
|  | | | **Anterior DTC** | | | **Posterior DTC** | | | **Anterior DTC** | | | **Posterior DTC** | | |  |
| **Exp’t#^3^** | **Strain^4^** | **Temp^5^** | **mutant/**  **total** | **%** | **95%C.I.^6^** | **mutant/**  **total** | **%** | **95%C.I.^6^** | **mutant/**  **total** | **%** | **95%C.I.^6^** | **mutant/**  **total** | **%** | **95%C.I^6^** | **hours**  **starved** |
| **TOTAL** | ***ngat-1(ev821)*** | **20^o^C** | **14/250** | **6%** | **3-9%** | **116/251** | **48%** | **40-55%** | **1/84** | **1%** | **0.1-7%** | **2/84** | **2%** | **1-9%** | **48** |
| **TOTAL** | ***mig-22(k141)*** | **20^o^C** | **122/313** | **39%** | **34-44%** | **219/304** | **72%** | **67-77%** | **26/176** | **15%** | **10-21%** | **58/173** | **34%** | **27-41%** | **48&51** |
| **TOTAL** | ***ev821; mig-22*** | **20^o^C** | **68/74** | **92%** | **83-97%** | **60/73** | **82%** | **71-90%** | **55/61** | **90%** | **79-96%** | **54/66** | **82%** | **70-90%** | **27** |
|  |  |  |  |  |  |  |  |  |  |  |  |  |  |  |  |
| **TOTAL** | ***ngat-1(ev840)*** | **20^o^C** | **1/342** | **0.3%** | **0.01-1%** | **241/551** | **44%** | **40-48%** | **4/238** | **2%** | **0.6-4%** | **6/237** | **3%** | **1-5%** | **43&50** |
| **TOTAL** | ***mig-22(k141)*** | **20^o^C** | **122/313** | **39%** | **34-44%** | **219/304** | **72%** | **67-77%** | **26/176** | **15%** | **10-21%** | **58/173** | **34%** | **27-41%** | **48&51** |
| **TOTAL** | ***ev840; mig-22*** | **20^o^C** | **203/210** | **97%** | **93-99%** | **185/204** | **91%** | **86-94%** | **58/61** | **95%** | **85-99%** | **56/61** | **92%** | **81-97%** | **50** |
|  |  |  |  |  |  |  |  |  |  |  |  |  |  |  |  |
| **TOTAL** | ***mig-17(k174)*** | **20^o^C** | **73/447** | **16%** | **13-20%** | **192/464** | **41%** | **37-46%** | **15/203** | **7%** | **4-12%** | **29/205** | **14%** | **10-20%** | **45&49** |
| **TOTAL** | ***mig-22(k141)*** | **20^o^C** | **122/313** | **39%** | **34-44%** | **219/304** | **72%** | **67-77%** | **26/176** | **15%** | **10-21%** | **58/173** | **34%** | **27-41%** | **48&51** |
| **12/3/19** | ***mig-22;mig-17*** | **20^o^C** | **69/74** | **93%** | **84-97%** | **59/75** | **79%** | **67-87%** | **85/110** | **77%** | **68-85%** | **52/105** | **50%** | **40-59%** | **48** |
|  |  |  |  |  |  |  |  |  |  |  |  |  |  |  |  |
| **TOTAL** | ***ngat-1(ev840)*** | **25^o^C** | **6/491** | **1%** | **0.5-3%** | **296/501** | **59%** | **55-63%** | **1/81** | **1%** | **0.1-8%** | **5/86** | **6%** | **2-18%** | **49** |
| **TOTAL** | ***mig-17(k174)*** | **25^o^C** | **77/385** | **20%** | **16-24%** | **267/390** | **68%** | **64-73%** | **3/57** | **5%** | **1-16%** | **5/57** | **9%** | **3-20%** | **25** |
| **TOTAL** | ***ngat-1; mig-17*** | **25^o^C** | **34/117** | **29%** | **21-38%** | **94/116** | **81%** | **72-87%** | **9/132** | **7%** | **3-13%** | **22/128** | **17%** | **11-25%** | **46** |

**Table S2A footnotes 1-6 as in Table S1A. Details of data contributing to summary Table S2A can be found in Table S2B.**

| **Table S2B: Penetrance of DTC phase 2 migration defects in double mutants of *ngat-1,* *mig-22(k141)* and *mig-17 (k174)* (raw data)** | | | | | | | | | | | | | | | |
| --- | --- | --- | --- | --- | --- | --- | --- | --- | --- | --- | --- | --- | --- | --- | --- |
|  | | | **CONTINUOUSLY WELL-FED^1^** | | | | | | **STARVED AND RE-FED^2^** | | | | | |  |
|  | | | **Anterior DTC** | | | **Posterior DTC** | | | **Anterior DTC** | | | **Posterior DTC** | | |  |
| **Exp’t#^3^** | **Strain^4^** | **Temp^5^** | **mutant/**  **total** | **%mut**  **-ant** | **95%C.I.^6^** | **mutant/**  **total** | **%mut**  **-ant** | **95%C.I.^6^** | **mutant/**  **total** | **%mut**  **-ant** | **95%C.I.^6^** | **mutant/**  **total** | **%mut**  **-ant** | **95%C.I^6^** | **hours starved** |
| **TOTAL** | ***ngat-1(ev821)*** | **20^o^C** | **14/250** | **6%** | **3-9%** | **116/251** | **48%** | **40-55%** |  |  |  |  |  |  |  |
| **3/21/19** | ***ngat-1(ev821)*** | **20^o^C** |  |  |  |  |  |  | **1/84** | **1%** | **0.1-7%** | **2/84** | **2%** | **1-9%** | **48** |
|  |  |  |  |  |  |  |  |  |  |  |  |  |  |  |  |
| **TOTAL** | ***mig-22(k141)*** | **20^o^C** | **122/313** | **39%** | **34-44%** | **219/304** | **72%** | **67-77%** |  |  |  |  |  |  |  |
| **4/11/24** | ***mig-22(k141)*** | **20^o^C** |  |  |  |  |  |  | **9/77** | **12%** | **6-22%** | **23/80** | **29%** | **19-40%** | **48** |
| **4/19/19** | ***mig-22(k141)*** | **20^o^C** |  |  |  |  |  |  | **17/99** | **17%** | **11-26%** | **35/93** | **38%** | **28-48%** | **51** |
| **TOTAL** | ***mig-22(k141)*** | **20^o^C** |  |  |  |  |  |  | **26/176** | **15%** | **10-21%** | **58/173** | **34%** | **27-41%** | **48&51** |
| **4/11/19** | ***mig-22(k141)*** | **20^o^C** |  |  |  |  |  |  | **0/83** | **0%** | **0-6%** | **2/84** | **2%** | **0.4-9%** | **Dauer** |
|  |  |  |  |  |  |  |  |  |  |  |  |  |  |  |  |
| **5/24/18** | ***ev821; mg-22*** | **20^o^C** | **53/58** | **91%** | **80-97%** | **45/57** | **74%** | **66-88%** |  |  |  |  |  |  |  |
| **5/25/18** | ***ev821; mg-22*** | **20^o^C** | **15/16** | **94%** | **72-99%** | **15/16** | **94%** | **72-99%** |  |  |  |  |  |  |  |
| **TOTAL** | ***ev821; mg-22*** | **20^o^C** | **68/74** | **92%** | **83-97%** | **60/73** | **82%** | **71-90%** |  |  |  |  |  |  |  |
| **5/27/18** | ***ev821; mg-22*** | **20^o^C** |  |  |  |  |  |  | **55/61** | **90%** | **79-96%** | **54/66** | **82%** | **70-90%** | **27** |
|  |  |  |  |  |  |  |  |  |  |  |  |  |  |  |  |
| **TOTAL** | ***ngat-1(ev840)*** | **20^o^C** | **1/342** | **0.3%** | **0.01-1%** | **241/551** | **44%** | **40-48%** |  |  |  |  |  |  |  |
| **3/21/19** | ***ngat-1(ev840)*** | **20^o^C** |  |  |  |  |  |  | **0/91** | **0%** | **0-5%** | **2/91** | **2%** | **1-8%** | **48** |
|  |  |  |  |  |  |  |  |  |  |  |  |  |  |  |  |
| **1/23/19** | ***ev840; mig-22*** | **20^o^C** | **64/66** | **97%** | **89-99%** | **54/61** | **89%** | **77-95%** |  |  |  |  |  |  |  |
| **2/5/19** | ***ev840; mig-22*** | **20^o^C** | **73/76** | **96%** | **88-99%** | **73/76** |  |  |  |  |  |  |  |  |  |
| **10/16/18** | ***ev840; mig-22*** | **20^o^C** | **66/68** |  |  | **58/67** | **87%** | **76-93%** |  |  |  |  |  |  |  |
| **TOTAL** | ***ev840; mig-22*** | **20^o^C** | **203/210** | **97%** | **93-99%** | **185/204** | **91%** | **86-94%** |  |  |  |  |  |  |  |
| **1/23/19** | ***ev840; mig-22*** | **20^o^C** |  |  |  |  |  |  | **58/61** | **95%** | **85-99%** | **56/61** | **92%** | **81-97%** | **50** |
|  |  |  |  |  |  |  |  |  |  |  |  |  |  |  |  |
| **TOTAL** | ***mig-17(k174)*** | **20^o^C** | **73/447** | **16%** |  | **192/464** | **41%** |  |  |  |  |  |  |  |  |
| **9/9/18** | ***mig-17(k174)*** | **20^o^C** |  |  |  |  |  |  | **3/57** |  |  | **5/57** | **9%** |  | **25** |
| **01/18/20** | ***mig-17(k174)*** | **20^o^C** |  |  |  |  |  |  | **10/90** | **11%** | **6-20%** | **11/93** | **12%** | **6-21%** | **49** |
| **7/4/20** | ***mig-17(k174)*** | **20^o^C** |  |  |  |  |  |  | **5/113** |  |  | **18/112** | **16%** |  | **45** |
| **TOTAL** | ***mig-17(k174)*** | **20^o^C** |  |  |  |  |  |  | **15/203** | **7%** | **4-12%** | **29/205** | **14%** | **10-20%** | **45&49** |
|  |  |  |  |  |  |  |  |  |  |  |  |  |  |  |  |
| **12/3/19** | ***mig-22; mig-17*** | **20^o^C** | **69/74** | **93%** | **84-97%** | **59/75** | **79%** | **67-87%** |  |  |  |  |  |  |  |
| **12/13/19** | ***mig-22; mig-17*** | **20^o^C** |  |  |  |  |  |  | **85/110** | **77%** | **68-85%** | **52/105** | **50%** | **40-59** | **48** |
|  |  |  |  |  |  |  |  |  |  |  |  |  |  |  |  |
| **TOTAL** | ***mig-17(k174)*** | **25^o^C** | **77/385** | **20%** | **16-24%** | **267/390** | **68%** | **64-73%** |  |  |  |  |  |  |  |
| **9/9/18** | ***mig-17(k174)*** | **25^o^C** |  |  |  |  |  |  | **3/57** | **5%** | **1-16%** | **5/57** | **9%** | **3-20%** | **25** |
|  |  |  |  |  |  |  |  |  |  |  |  |  |  |  |  |
| **TOTAL** | ***ngat-1(ev840)*** | **25^o^C** | **6/491** | **1%** | **0.5-3%** | **296/501** | **59%** | **55-63%** |  |  |  |  |  |  |  |
| **4/4/19** | ***ngat-1(ev840)*** | **25^o^C** |  |  |  |  |  |  | **1/81** | **1%** | **0.1-8%** | **5/86** | **6%** | **2-18%** | **49** |
|  |  |  |  |  |  |  |  |  |  |  |  |  |  |  |  |
| **2/23/20** | ***ev840; mig-17*** | **25^o^C** | **9/50** |  |  | **42/51** |  |  |  |  |  |  |  |  |  |
| **7/15/20** | ***ev840; mig-17*** | **25^o^C** | **25/67** |  |  | **52/65** |  |  |  |  |  |  |  |  |  |
| **TOTAL** | ***ev840; mig-17*** | **25^o^C** | **34/117** | **29%** | **21-38%** | **94/116** | **81%** | **72-87%** |  |  |  |  |  |  |  |
| **7/17/20** | ***ev840; mig-17*** | **25^o^C** |  |  |  |  |  |  | **9/132** | **7%** | **3-13%** | **22/128** | **17%** | **11-25%** | **46** |

**Table S2B footnotes as in Table S1A.**
