# Supplementary material for: Distal tip cell migration mutants of Caenorhabditis elegans are rescued by bioequivalent outputs from chondroitin and N-glycosylation pathways
Source: J Biol Chem. 2025 Nov 4;301(12):110895. doi: 10.1016/j.jbc.2025.110895 (PMC12721165; doi:10.1016/j.jbc.2025.110895)
Supplement: Table S3 [file mmc3.docx]

| **Table S3: Rescue of glycosylation mutant DTC migration defects by feeding GlcNAc** | | | | | | | | | | | | | | | |
| --- | --- | --- | --- | --- | --- | --- | --- | --- | --- | --- | --- | --- | --- | --- | --- |
|  | | | **CONTINUOUSLY WELL-FED, No GlcNAc^1^** | | | | | | **CONTINUOUSLY WELL-FED, + 7-10 mM GlcNAc ^2^** | | | | | |  |
|  | | | **Anterior DTC** | | | **Posterior DTC** | | | **Anterior DTC** | | | **Posterior DTC** | | |  |
| **Exp’t#^3^** | **Strain^4^** | **Temp^5^** | **mutant/**  **total** | **%** | **95%C.I.^6^** | **mutant/**  **total** | **%** | **95%C.I.^6^** | **mutant/**  **total** | **%** | **95%C.I.^6^** | **mutant/**  **total** | **%** | **95%C.I^6^** | **[GlcNAc]^7^** |
| **TOTAL** | ***ngat-1(ev821)*** | **25^o^C** | **14/416** | **3%** | **2-6%** | **310/439** | **71%** | **66-75%** |  |  |  |  |  |  |  |
| **8/12/18** | ***ngat-1(ev821)*** | **25^o^C** |  |  |  |  |  |  | **1/47** | **2%** | **0.1-13%** | **3/50** | **6%** | **1.5-8%** | **7 mM** |
|  |  |  |  |  |  |  |  |  |  |  |  |  |  |  |  |
| **TOTAL** | ***ngat-1(ev840)*** | **20^o^C** | **1/342** | **0.3%** | **0.01-1%** | **241/551** | **44%** | **40-48%** |  |  |  |  |  |  |  |
| **4/28/19** | ***ngat-1(ev840)*** | **20^o^C** |  |  |  |  |  |  | **2/59** | **3%** | **1-13%** | **3/59** | **5%** | **1-15%** | **10 mM** |
| **2/5/19** | ***ngat-1(ev840)*** | **20^o^C** |  |  |  |  |  |  | **0/75** | **0%** | **0-6%** | **6/77** | **8%** | **3-17%** | **10 mM** |
| **4/28/19** | ***ngat-1(ev840)*** | **20^o^C** |  |  |  |  |  |  | **0/36** | **0%** | **0-12%** | **2/36** | **6%** | **1-20%** | **10mM** |
| **TOTAL** | ***ngat-1(ev840)*** | **20^o^C** |  |  |  |  |  |  | **2/170** | **1%** | **0.2-5%** | **11/172** | **6%** | **3-11%** | **10mM** |
|  |  |  |  |  |  |  |  |  |  |  |  |  |  |  |  |
| **TOTAL** | ***ngat-1(ev840)*** | **25^o^C** | **6/491** | **1%** | **0.5-3%** | **296/501** | **59%** | **55-63%** |  |  |  |  |  |  |  |
| **9/3/18** | ***ngat-1(ev840)*** | **25^o^C** |  |  |  |  |  |  | **1/64** | **2%** | **0.1-10%** | **3/69** | **4%** | **1-13%** | **10mM** |
| **11/21/20** | ***ngat-1(ev840)*** | **25^o^C** |  |  |  |  |  |  | **2/125** | **2%** | **0.4-6%** | **24/125** | **19%** | **13-27%** | **10 mM** |
| **TOTAL** | ***ngat-1(ev840)*** | **25^o^C** |  |  |  |  |  |  | **3/189** | **1.5%** | **0.5-5%** | **27/194** | **14%** | **10-20%** | **10mM** |
|  |  |  |  |  |  |  |  |  |  |  |  |  |  |  |  |
| **TOTAL** | ***mig-22(k141)*** | **20^o^C** | **122/313** | **39%** | **34-44%** | **219/304** | **72%** | **67-77%** |  |  |  |  |  |  |  |
| **8/8/18** | ***mig-22(k141)*** | **20^o^C** |  |  |  |  |  |  | **11/46** | **24%** | **13-39%** | **31/47** | **66%** | **51-79%** | **7 mM** |
|  |  |  |  |  |  |  |  |  |  |  |  |  |  |  |  |
| **TOTAL** | ***mig-17(k174)*** | **20^o^C** | **73/447** | **16%** | **13-20%** | **192/464** | **41%** | **37-46%** |  |  |  |  |  |  |  |
| **1/05/19** | ***mig-17(k174)*** | **20^o^C** |  |  |  |  |  |  | **9/29** | **31%** | **16-51%** | **16/42** | **38%** | **24-54%** | **10 mM** |
|  |  |  |  |  |  |  |  |  |  |  |  |  |  |  |  |
| **TOTAL** | ***mig-17(k174)*** | **25^o^C** | **77/385** | **20%** | **16-24%** | **267/390** | **68%** | **64-73%** |  |  |  |  |  |  |  |
| **12/8/18** | ***mig-17(k174)*** | **25^o^C** |  |  |  |  |  |  | **1/41** | **2%** | **0.1-14%** | **21/46** | **46%** | **31-61%** | **7mM** |
| **12/29/18** | ***mig-17(k174)*** | **25^o^C** |  |  |  |  |  |  | **5/57** | **9%** | **3-19%** | **39/52** | **75%** | **61-86%** | **7 mM** |
|  |  |  |  |  |  |  |  |  | **5/98** | **5%** | **2-12%** | **60/98** | **61%** | **51-71%** | **7mM** |
|  |  |  |  |  |  |  |  |  |  |  |  |  |  |  |  |
| **TOTAL** | ***ev840; mig-22*** | **20^o^C** | **203/210** | **97%** | **93-99%** | **185/204** | **91%** | **86-94%** |  |  |  |  |  |  |  |
| **2/5/19** | ***ev840; mig-22*** | **20^o^C** |  |  |  |  |  |  | **78/81** | **96%** | **89-99%** | **76/81** | **94%** | **86-98%** | **10 mM** |
| **1/23/19** | ***ev840; mig-22*** | **20^o^C** |  |  |  |  |  |  | **58/61** | **95%** | **85-99%** | **55/58** | **95%** | **85-99%** | **10mM** |
|  |  |  |  |  |  |  |  |  |  |  |  |  |  |  |  |
| **TOTAL** | ***mgat-1* triple** | **25^o^C** | **6/310** | **2%** | **1-4%** | **173/323** | **54%** | **48-59%** |  |  |  |  |  |  |  |
| **4/13/17** | ***mgat-1 Itriple*** | **25^o^C** | **N.D.** |  |  | **57/97** | **59%** | **48-69%** |  |  |  | **16/87** | **18%** | **11-28%** | **7mM** |
| **TOTAL** | ***mgat-1* triple** | **25^o^C** | **6/310** | **2%** | **1-4%** | **173/323** | **54%** | **48-59%** |  |  |  |  |  |  |  |

**Table S3 footnotes 1-6 as in Table S1A. ^7^ Concentrations of GlcNAc on seeded plates was changed when calculation error was noted.**
