# Supplementary material for: Distal tip cell migration mutants of Caenorhabditis elegans are rescued by bioequivalent outputs from chondroitin and N-glycosylation pathways
Source: J Biol Chem. 2025 Nov 4;301(12):110895. doi: 10.1016/j.jbc.2025.110895 (PMC12721165; doi:10.1016/j.jbc.2025.110895)
Supplement: Table S4 [file mmc4.docx]

| **Table S4: Rescue by gain-of function mutations in *gfat-1* and in *mig-22*** | | | | | | | | |
| --- | --- | --- | --- | --- | --- | --- | --- | --- |
|  | | | **CONTINUOUSLY WELL-FED^1^  ( *AL)*** | | | | | |
|  | | | **Anterior DTC** | | | **Posterior DTC** | | |
| **Exp’t#^3^** | **Strain^4^** | **Temp^5^** | **mutant/**  **total** | **%** | **95%C.I.^6^** | **mutant/**  **total** | **%** | **95%C.I.^6^** |
| **6/12/24** | ***gfat-1(dh468)*** | **20^o^C** | **0/26** | **0%** | **0-16%** | **0/42** | **0%** | **0-10%** |
| **6/13/24** | ***gfat-1(dh468)*** | **20^o^C** | **0/36** | **0%** | **0-12%** | **3/36** | **8%** | **2-24%** |
| **TOTAL** | ***gfat-1(dh468)*** | **20^o^C** | **0/62** | **0%** | **0-7%** | **3/78** | **4%** | **1-11%** |
|  |  |  |  |  |  |  |  |  |
| **1/9/25** | ***mig-22(k185)*** | **20^o^C** | **0/17** |  |  | **0/17** |  |  |
| **1/10/25** | ***mig-22(k185)*** | **20^o^C** | **0/43** | **0%** | **0-10%** | **2/53** | **4%** | **0.6-14%** |
| **TOTAL** | ***mig-22(k185)*** | **20^o^C** | **0/60** | **0%** | **0-7.5%** | **2/70** | **3%** | **0.5-11%** |
| **1/10/25** | ***mig-22(k185)*** | **20^o^C** | **0/43** | **0%** | **0-10%** | **2/53** | **4%** | **0.7-14%** |
| **TOTAL** | ***mig-22(k185)*** | **20^o^C** | **0/60** | **0%** | **0-7%** | **2/70** | **3%** | **0.5-11%** |
|  |  |  |  |  |  |  |  |  |
| **TOTAL** | ***ngat-1(ev840)*** | **20^o^C** | **1/342** | **0.3%** | **0.01-1%** | **241/551** | **44%** | **40-48%** |
| **TOTAL** | ***mig-22(k185)*** | **20^o^C** | **0/60** | **0%** | **0-7%** | **2/70** | **3%** | **0.5-11%** |
| **6/30/24** | ***ev840; k185*** | **20^o^C** | **4/102** | **4%** | **1-10%** | **1/107** | **1%** | **.05-6%** |
|  |  |  |  |  |  |  |  |  |
| **TOTAL** | ***ngat-1(ev840)*** | **25^o^C** | **14/416** | **3%** | **2-6%** | **310/439** | **71%** | **67-79%** |
| **1/20/25** | ***mig-22(k185)*** | **25^o^C** | **2/60** | **3%** | **0.6-12%** | **3/73** | **4%** | **1-12%** |
| **1/27/25** | ***ev840; k185*** | **25^o^C** | **2/53** | **4%** | **0.7-14%** | **1/62** | **2%** | **0.1-10%** |
|  |  |  |  |  |  |  |  |  |
| **TOTAL** | ***mig-17(k174)*** | **20^o^C** | **73/447** | **16%** | **13-20%** | **192/464** | **41%** | **37-46%** |
| **TOTAL** | ***mig-22(k185)*** | **20^o^C** | **0/60** | **0%** | **0-7%** | **2/70** | **3%** | **0.5-11%** |
| **1/6/25** | ***k185; k174*** | **20^o^C** | **0/51** | **0%** | **0-8%** | **2/54** | **4%** | **0.6-13%** |
|  |  |  |  |  |  |  |  |  |
| **TOTAL** | ***mig-22(k141)*** | **20^o^C** | **122/313** | **39%** | **34-44%** | **219/304** | **72%** | **67-77%** |
| **TOTAL** | ***gfat-1(dh468)*** | **20^o^C** | **0/62** | **0%** | **0-7%** | **3/78** | **4%** | **1-11%** |
| **6/7/24** | ***dh468; k141*** | **20^o^C** | **1/63** | **2%** | **0.1-10%** | **1/84** | **1%** | **0.1-7%** |
|  |  |  |  |  |  |  |  |  |
| **TOTAL** | ***mig-17(k174)*** | **20^o^C** | **73/447** | **16%** | **13-20%** | **192/464** | **41%** | **37-46%** |
| **TOTAL** | ***gfat-1(dh468)*** | **20^o^C** | **0/62** | **0%** | **0-7%** | **3/78** | **4%** | **1-11%** |
| **7/18/24** | ***dh468; k174*** | **20^o^C** | **0/96** | **0%** | **0-5%** | **20/93** | **22%** | **14-31%** |

**Table S4 footnotes 1-6 as in Table S1A.**
